# Discovery of Zharp1-163 as a dual inhibitor of ferroptosis and necroptosis for the treatment of inflammatory disorders and kidney injury

**DOI:** 10.1038/s41420-025-02693-5

**Published:** 2025-08-28

**Authors:** Yuting Ji, Shujing Du, Jingjing Li, Haikuo Ma, Xinhui Wang, Yongjin Hao, Zhanhui Li, Haohao Lu, Hao Liu, Chengkui Yang, Xiaohu Zhang, Sudan He

**Affiliations:** 1 State Key Laboratory of Common Mechanism Research for Major Diseases, and Key Laboratory of Synthetic Biology Regulatory Elements, Suzhou Institute of Systems Medicine, Chinese Academy of Medical Sciences & Peking Union Medical College, Suzhou, 215123 Jiangsu China; 2 School of Life Science and Technology, China Pharmaceutical University, Nanjing, Jiangsu China; 3 Jiangsu Key Laboratory of Neuropsychiatric Diseases and College of Pharmaceutical Sciences, Soochow University, Suzhou, 215123 China; 4 School of Chemistry, Chemical Engineering and Life Science, Wuhan University of Technology, Wuhan, 430070 Hubei People’s Republic of China

**Keywords:** Acute kidney injury, Necroptosis

## Abstract

Dysregulation of cell death plays a critical role in the onset and progression of numerous human diseases. Distinct forms of regulated cell death, such as necroptosis and ferroptosis, have been implicated in the pathogenesis of various conditions, including neurodegenerative disorders and acute kidney injury. Strategies that concurrently target both necroptosis and ferroptosis present significant potential for improving therapeutic outcomes. In this study, we identified Zharp1-163 as a dual inhibitor of ferroptosis and necroptosis in both human and mouse species. Zharp1-163 effectively blocks ferroptosis by reducing reactive oxygen species (ROS) levels and inhibits necroptosis by potently and selectively targeting RIPK1 kinase activity. In vivo, Zharp1-163 markedly attenuated TNF-α-induced systemic inflammatory syndrome, including the prevention of TNF-α-induced mortality and hypothermia in mice. Notably, Zharp1-163 significantly alleviated acute kidney injury associated with both necroptosis and ferroptosis in models induced by cisplatin treatment and ischemia-reperfusion. Collectively, our findings establish Zharp1-163 as a dual-action inhibitor capable of effectively suppressing both ferroptosis and necroptosis. These findings highlight its great potential in the treatment of diseases associated with these cell death pathways, such as kidney disease.

## Introduction

Cell death is the final stage of the cell life cycle and serves as a crucial mechanism for organisms to maintain tissue homeostasis. On the basis of the preservation of cell membrane integrity during the cell death process, cell death can be classified into two major categories: nonlytic cell death and lytic cell death [1]. Apoptosis is a well-known form of nonlytic cell death [2], which is characterized by an intact cell membrane, preventing leakage of cellular contents into the extracellular space. As a result, this type of cell death usually does not provoke an inflammatory response. In contrast, necrosis is a form of lytic cell death characterized by the loss of cell membrane integrity, leading to the release of cellular contents into the extracellular environment, which induces inflammatory responses and immune activation [3–5]. This category of cell death includes necroptosis and ferroptosis, among others. Excessive or aberrant lytic cell death can cause tissue damage and chronic inflammation, contributing to diseases such as neurodegenerative disorders, cardiovascular diseases, cancer, and acute organ injuries. Targeting relevant cell death pathways represents a promising therapeutic strategy for ameliorating associated diseases and enhancing treatment efficacy.

Ferroptosis is a form of regulated necrosis characterized by the accumulation of iron, lipid peroxidation and the production of reactive oxygen species (ROS) [5, 6]. It arises from the dysregulation of interconnected metabolic pathways: disrupted lipid metabolism leads to peroxidation of polyunsaturated fatty acid (PUFA)-containing phospholipids, whereas iron overload via the Fenton reaction exacerbates oxidative damage [7]. Key regulatory nodes include the System Xc^−^-GSH-GPX4 axis, which suppresses lipid peroxidation [5, 8, 9], and iron metabolism, which is mediated by ferritinophagy and transferrin receptors [10–13]. Mechanistically, ferroptosis execution involves membrane rupture driven by peroxidation-derived pore-forming radicals and compromised antioxidant defences. Ferroptosis is implicated in multiple pathologies, including cancer, neurodegenerative disorders, and ischemia-reperfusion injury [14–17]. Therapeutic modulation of ferroptosis regulators shows promise for disease-specific interventions.

Necroptosis is a form of regulated necrosis that is distinct from apoptosis and is characterized by cell swelling, plasma membrane rupture, and the release of inflammatory intracellular components [3]. It is triggered when apoptosis is inhibited and mediated by receptor-interacting protein kinases (RIPK1/RIPK3) and mixed-lineage kinase domain-like pseudokinase (MLKL) [3, 18, 19]. Activation through death receptors initiates the interaction of RIPK1 and RIPK3 via their RIP homotypic interaction motif (RHIM), leading to their phosphorylation and necrosome formation [20–22]. Subsequently, RIPK3 activates MLKL, leading to MLKL oligomerization and translocation to the plasma membrane, ultimately causing membrane rupture and necroptosis [18, 19, 23–26]. Dysregulated necroptosis contributes to pathologies such as neurodegenerative diseases, ischemic injuries, and viral infections [3, 27, 28]. Therefore, targeting necroptosis signaling pathways represents a promising therapeutic strategy for diseases associated with necroptosis.

Studies have demonstrated that ferroptosis and necroptosis can occur in the same disease state, such as kidney disease, acute myocardial infarction, stroke and cancer [29–33]. Ferrostatin-1 and liproxstatin-1, radical-trapping antioxidants, are widely used to inhibit ferroptosis and have been shown to attenuate acute kidney injury [34, 35]. Necrostatin-1 (Nec-1), an extensively studied RIPK1 kinase inhibitor, prevents necroptosis [3] and exhibits inhibition of cytokine-driven tubular cell death and kidney injury [36–38]. Considering the involvement of ferroptosis and necroptosis in some diseases, the development of dual inhibitors capable of simultaneously targeting both pathways could offer substantial therapeutic potential.

In our study, we identified Zharp1-163 as a potent dual inhibitor of ferroptosis and necroptosis in human and mouse cells. Zharp1-163 suppresses reactive oxygen species (ROS) levels to inhibit ferroptosis and acts as a selective RIPK1 kinase inhibitor to block necroptosis. Treatment with Zharp1-163 in mice can alleviate TNF-α-induced RIPK1-driven systemic inflammatory response syndrome (SIRS). Notably, Zharp1-163 also significantly mitigated acute kidney injury linked to both necroptosis and ferroptosis in models induced by ischemia-reperfusion and cisplatin treatment. These findings highlight Zharp1-163 as a dual inhibitor of ferroptosis and necroptosis and suggest the potential of Zharp1-163 as a starting point for the development of new approaches to treat associated diseases.

## Results

### Zharp1-163 is a potent dual inhibitor of ferroptosis and necroptosis

**Fig. 1:**
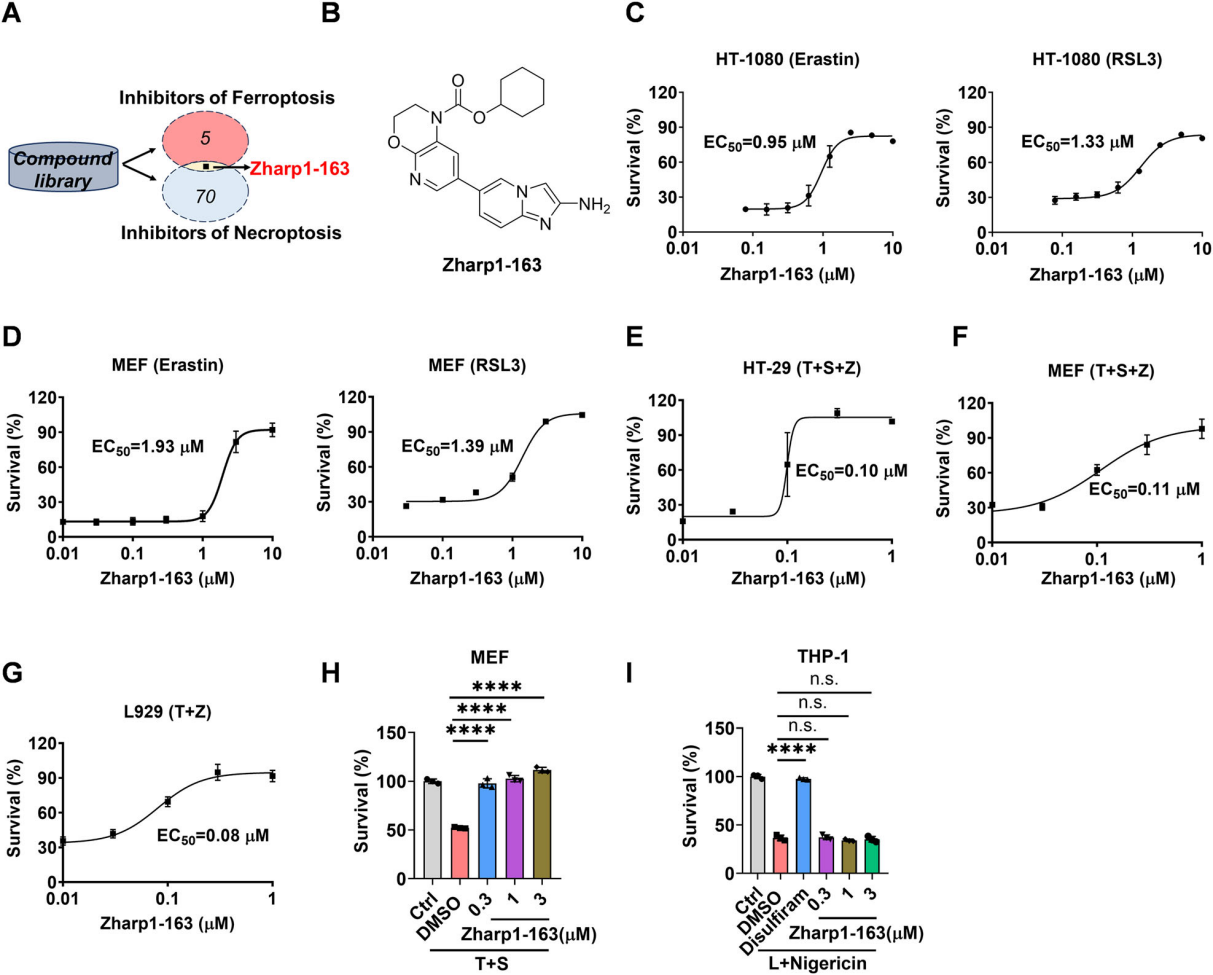
Zharp1-163 is a potent dual inhibitor of ferroptosis and necroptosis. **A** Schematic representation of the screening process for dual inhibitors of ferroptosis (Survival exceeds 50%) and necroptosis (Survival exceeds 50%). **B** Chemical structure of Zharp1-163. **C** HT-1080 cells were pretreated with the indicated concentrations of Zharp1-163 for 2 h and then treated with erastin or RSL3 for 16 h. Cell viability was assessed by measuring ATP levels. **D** MEFs were pretreated with the indicated concentrations of Zharp1-163 for 2 h and then treated with erastin or RSL3 for 16 h. Cell viability was assessed by measuring ATP levels. **E** Dose–response curve and EC_50_ values for the effects of Zharp1-163 on TNF-induced necroptosis in HT-29 cells. The cells were pretreated with the indicated concentrations of Zharp1-163 for 2 h, followed by treatment with T (40 ng/ml), S (100 nM) and Z (20 μM) for 16 h. Cell viability was assessed by measuring ATP levels. **F** MEFs were pretreated with the indicated concentrations of Zharp1-163 for 2 h, followed by treatment with T (40 ng/ml), S (100 nM) and Z (20 μM) for 12 h. Cell viability was assessed by measuring ATP levels. **G** L929 cells were pretreated with the indicated concentrations of Zharp1-163 for 2 h, followed by treatment with T (40 ng/ml) and Z (20 μM) for 12 h. Cell viability was assessed by measuring ATP levels. **H** MEFs were pretreated with the indicated concentrations of Zharp1-163 for 2 h prior to treatment with T (40 ng/ml) and S (100 nM) for 24 h. Cell viability was assessed by measuring ATP levels. **I** THP-1 cells were pretreated with the indicated concentrations of Zharp1-163 or 5 μM disulfiram (positive control) for 2 h and then treated with LPS (1 µg/ml) for 4 h, followed by nigericin (10 μM) for 2 h. Cell viability was assessed by measuring ATP levels. T, TNF-α; S, Smac mimetic; Z, z-VAD; L, lipopolysaccharide. The data are presented as the mean ± SD of triplicate samples. n.s., not significant; ****P < 0.0001.

To identify new inhibitors of ferroptosis and necroptosis, we screened a custom-made small-molecule library of approximately 207 potential inhibitors that target ferroptosis and necroptosis. Human colon cancer HT-29 cells were treated with these compounds for 2 h before being exposed to RSL3 to induce ferroptosis. Moreover, HT-29 cells were treated with these compounds for 2 h prior to treatment with necroptotic stimuli (TNFα, Smac mimetic and z-VAD), which are widely used to trigger TNF-induced necroptosis [18]. Zharp1-163 was identified as a dual inhibitor that effectively targeted both ferroptosis and necroptosis (Fig. 1A, B and S1). We found that Zharp1-163 significantly inhibited erastin- or RSL3-induced ferroptosis in HT-1080 cells (Fig. 1C). We further investigated the effect of Zharp1-163 on ferroptosis in MEFs and demonstrated the effective inhibition of this process (Fig. 1D). HT-29 cells were pretreated with Zharp1-163 for 2 h, followed by treatment with TNFα, Smac mimetic, and z-VAD, which are known to activate TNF-mediated necroptosis. Zharp1-163 blocked TNF-α-induced necroptosis in HT-29 cells, with an EC_50_ of approximately 0.10 μM (Fig. 1E). Zharp1-163 also efficiently inhibited TNF-mediated necroptosis, with EC_50_ values of approximately 0.11 μM in MEFs and 0.08 μM in mouse fibroblast L929 cells (Fig. 1F, G). We further assessed the effects of Zharp1-163 on apoptosis and pyroptosis in various cell lines. We found that Zharp1-163 inhibited apoptosis induced by TNFα plus Smac mimetic in MEFs (Fig. 1H). However, Zharp1-163 did not affect pyroptosis in THP-1 cells (Fig. 1I). These results reveal that Zharp1-163 is a potent dual inhibitor of ferroptosis and necroptosis in both human and mouse cells.

### Zharp1-163 suppresses lipid ROS peroxidation to inhibit ferroptosis

**Fig. 2:**
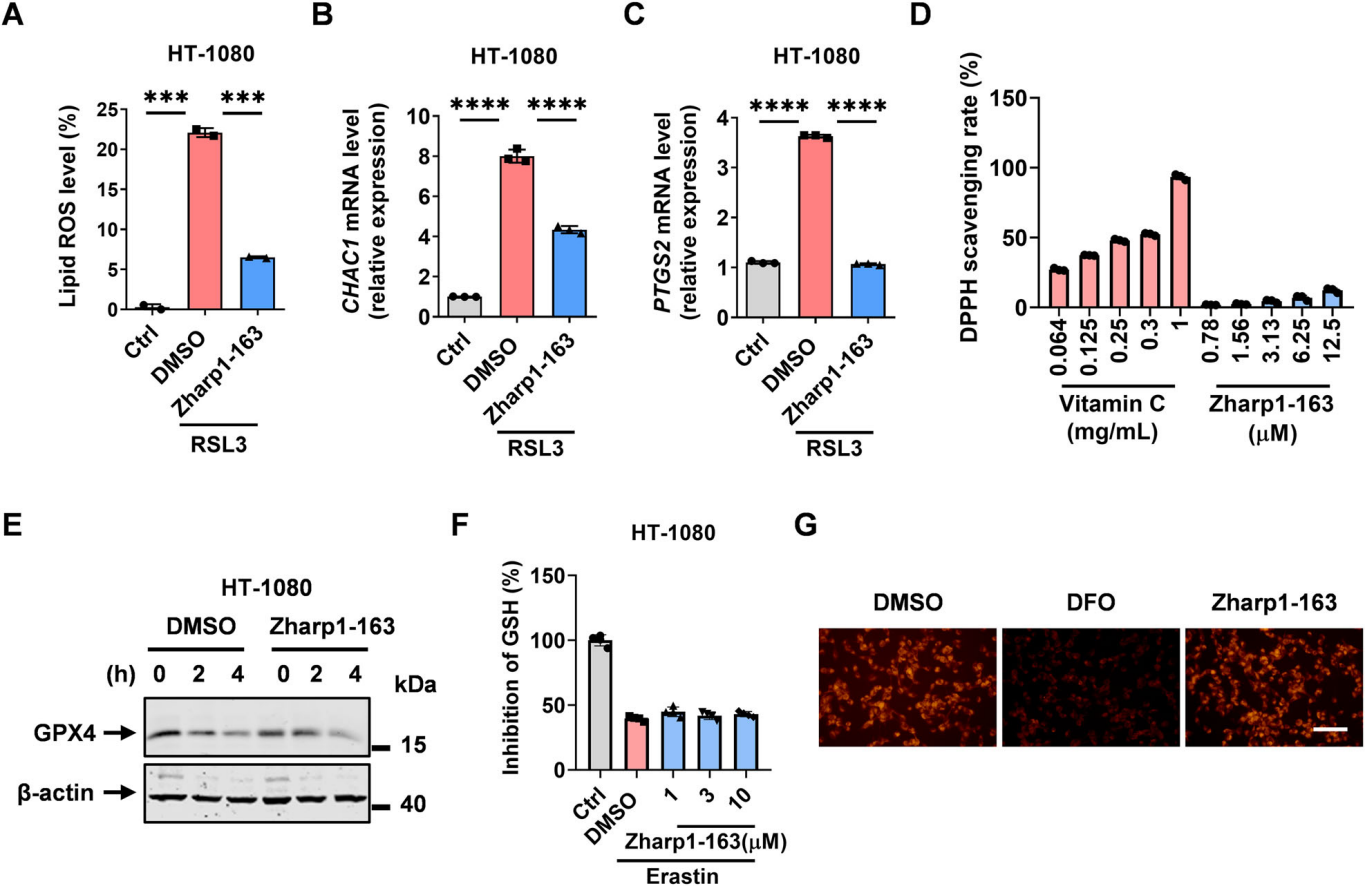
Zharp1-163 suppresses lipid ROS peroxidation to inhibit ferroptosis. **A** The ROS levels in HT-1080 cells were measured after pretreatment with DMSO or Zharp1-163 for 2 h, followed by induction with RSL3 for 5 h. **B**, **C** The levels of *CHAC1* and *PTGS2* mRNA expression were measured in HT-1080 cells pretreated with DMSO or Zharp1-163 for 2 h, followed by induction with RSL3 (**B**, **C**). **D** DPPH scavenging rates were detected after treatment with the indicated concentrations of vitamin C or Zharp1-163. **E** HT-1080 cells were treated with DMSO or Zharp1-163 for 2 h prior to treatment with erastin for different durations. The cells were collected, and GPX4 expression was analyzed via Western blotting. **F** GSH levels in HT-1080 cells were analyzed after pretreatment with the indicated concentrations of Zharp1-163 for 2 h, followed by induction with erastin. **G** Representative fluorescence microscopy images of HT-1080 cells treated with DFO (100 μM) or Zharp1-163 (10 μM), as assessed by the FerroOrange testing assay. Scale bar, 500 μm. The data are presented as the mean ± SD of triplicate samples. ***P < 0.001; ****P < 0.0001.

After identifying Zharp1-163 as a ferroptosis inhibitor, we further explored the molecular mechanisms through which it exerts its anti-ferroptosis effects. It is well understood that ferroptosis is an iron-dependent form of regulated cell death driven by lipid peroxidation and oxidative stress. We observed an increase in lipid ROS production during ferroptosis induced by RSL3, which was significantly reduced by Zharp1-163 (Fig. 2A). This suggests that Zharp1-163 exerts an inhibitory effect on RSL3-induced lipid ROS production during ferroptosis. The GSH-degrading enzyme (*CHAC1*) and prostaglandin-endoperoxide synthase 2 (*PTGS2*) are established biomarkers of ferroptosis, and their upregulation reflects lipid peroxidation and cellular oxidative stress responses [8, 39]. Consistently, Zharp1-163 significantly reduced the induction of both *CHAC1* and *PTGS2* in response to RSL3 (Fig. 2B, C). To elucidate whether the ferroptosis-inhibiting effect of Zharp1-163 is due to a potential direct antioxidative property, we conducted a radical reaction using 2,2-Diphenyl-1-picrylhydrazyl (DPPH) [40]. In this experiment, vitamin C acted as a radical scavenger, whereas Zharp1-163 displayed no observable antioxidant activity (Fig. 2D). The System Xc^−^-GSH-GPX4 axis is a key antioxidant system in ferroptosis. Zharp1-163 had no effects on GPX4 downregulation and GSH depletion during ferroptosis, ruling out a functional impact of Zharp1-163 on GPX4 (Fig. 2E, F). The iron chelator deferoxamine (DFO) is well-known to block Fe²⁺ accumulation. We observed that Zharp1-163 did not affect Fe²⁺ accumulation during ferroptosis (Fig. 2G). Collectively, these results indicate that Zharp1-163 inhibits ferroptosis through the suppression of lipid ROS peroxidation.

### Zharp1-163 blocks the cellular activation of RIPK1, RIPK3 and MLKL upon necroptotic stimulation

**Fig. 3:**
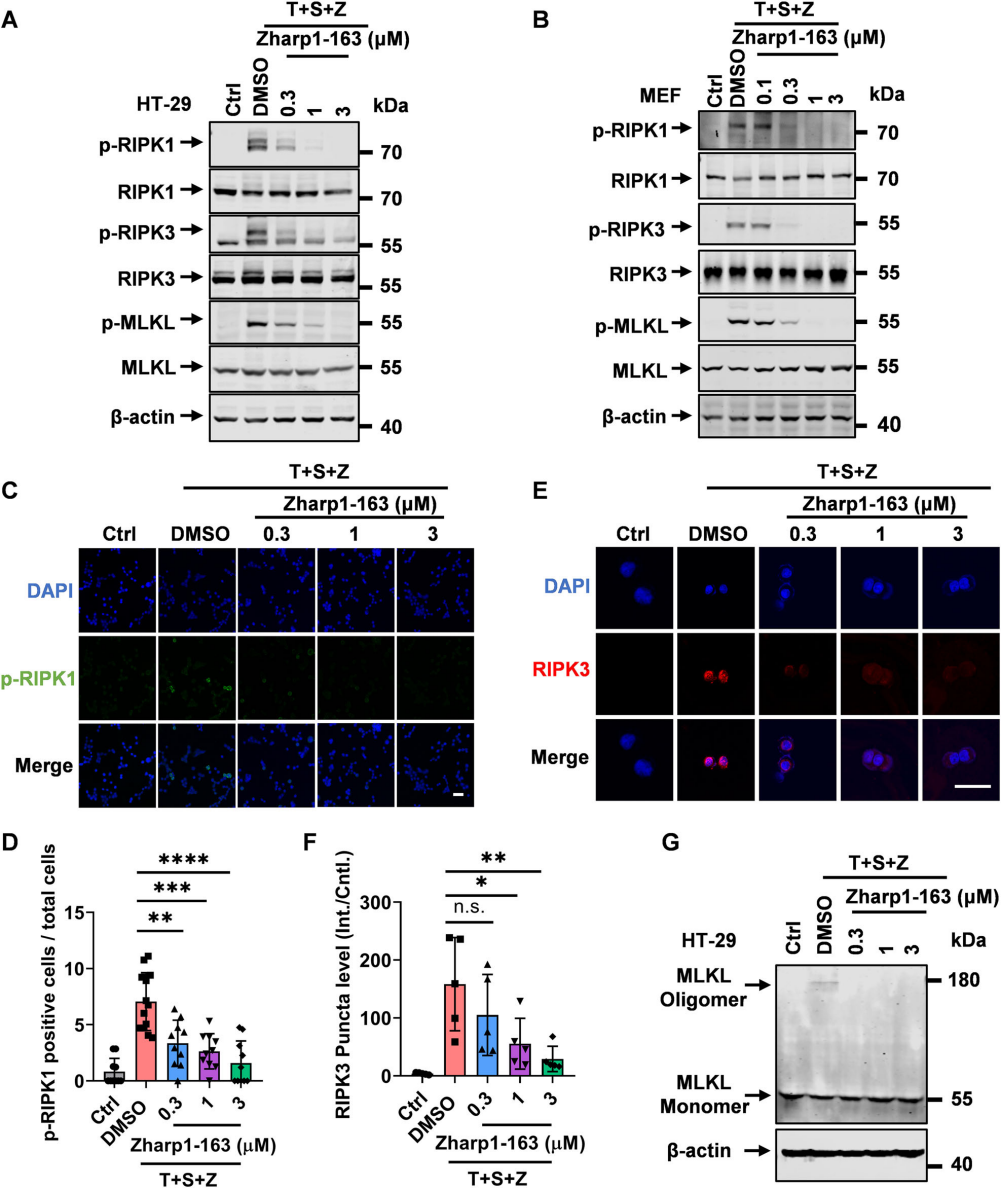
Zharp1-163 blocks the cellular activation of RIPK1, RIPK3 and MLKL upon necroptotic stimulation. **A**, **B** HT-29 and MEF cells were pretreated with DMSO or the indicated concentrations of Zharp1-163 for 2 h, followed by treatment with T (40 ng/ml), S (100 nM) and Z (20 μM) for 8 h. Cell lysates were harvested and then subjected to Western blot analysis for the phosphorylation of RIPK1, RIPK3, and MLKL. T, TNF-α; S, Smac mimetic; Z, z-VAD (**A**, **B**). **C**, **D** Effects of Zharp1-163 on RIPK1 phosphorylation. HT-29 cells were pretreated with Zharp1-163 for 2 h, followed by treatment with T (40 ng/ml), S (100 nM) and Z (20 μM) for an additional 8 h. The phosphorylation of RIPK1 was detected using immunofluorescence and subsequently quantified and analyzed (**C**, **D**). Scale bar, 25 μm. **E**, **F** Effects of Zharp1-163 on the formation of RIPK3 puncta. Prior to the addition of T (40 ng/ml), S (100 nM) and Z (20 μM) for an additional 8 h, HT-29 cells stably expressing Flag-tagged RIPK3 were preincubated with the specified compounds for 2 h (**E**, **F**). Scale bar, 25 μm. **G** Effects of Zharp1-163 on the oligomerization of MLKL. HT-29 cells were pretreated with the indicated concentrations of Zharp1-163 for 2 h, followed by treatment with T (40 ng/ml), S (100 nM) and Z (20 μM) for an additional 8 h. The data presented are representative of three independent experiments. The data are presented as the mean ± SD. n.s., not significant; *P < 0.05; **P < 0.01; ***P < 0.001; ****P < 0.0001.

Given that Zharp1-163 is a novel inhibitor of necroptosis, we investigated the molecular mechanism underlying its ability to inhibit necroptosis. RIPK1, RIPK3, and MLKL are activated during TNF-induced necroptosis, as evidenced by their phosphorylation. We examined the effects of Zharp1-163 on the phosphorylation of RIPK1, RIPK3, and MLKL in response to necroptotic stimuli. Treatment with Zharp1-163 eliminated the phosphorylation of RIPK1, RIPK3, and MLKL in human HT-29 cells (Fig. 3A). Consistently, Zharp1-163 blocked the phosphorylation of RIPK1, RIPK3, and MLKL in MEFs (Fig. 3B). We found that Zharp1-163 significantly inhibited RIPK1 phosphorylation in HT-29 cells (Fig. 3C, D). RIPK3 activation leads to puncta formation during necroptosis, and we observed that Zharp1-163 treatment prevented the generation of RIPK3 puncta (Fig. 3E, F). MLKL oligomerization is a crucial step in necroptosis, leading to cell membrane rupture and necrosis. We found that Zharp1-163 treatment prevented MLKL oligomerization (Fig. 3G). These results show that Zharp1-163 inhibits the cellular activation of RIPK1, RIPK3 and MLKL in response to necroptotic stimulation.

### Zharp1-163 is a selective inhibitor of RIPK1 kinase activity

**Fig. 4:**
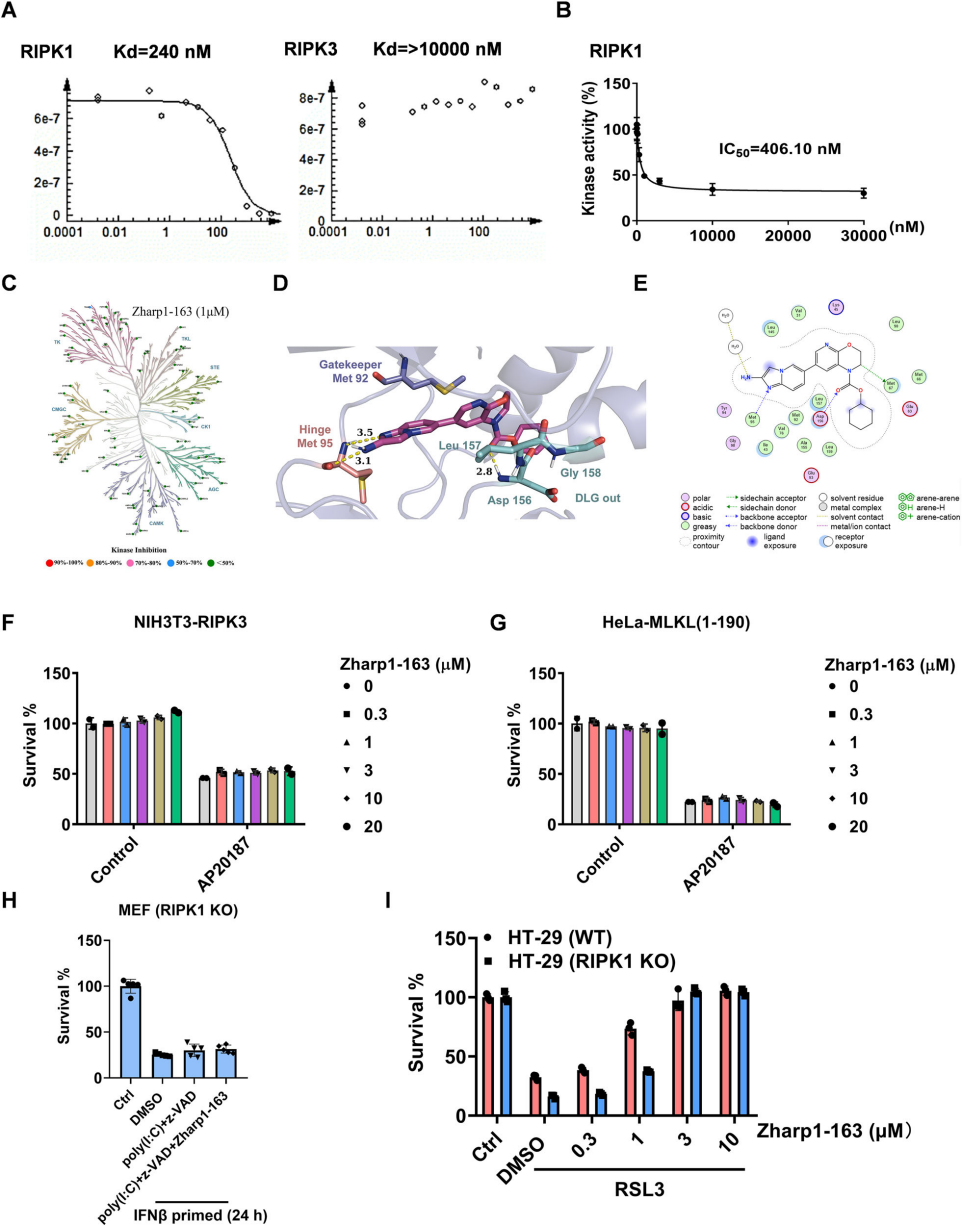
Zharp1-163 is a selective inhibitor of RIPK1 kinase activity. **A** Binding affinity curve of Zharp1-163 with RIPK1 and RIPK3. **B** In an in vitro kinase activity assay, recombinant RIPK1 was incubated with Zharp1-163, after which RIPK1 kinase activity was determined by measuring ATP levels via a luciferase reporter assay system. **C** The kinome selectivity of Zharp1-163 was assessed by establishing a screening model for the evaluation of the selectivity of 81 kinase targets, and the inhibition of Zharp1-163 on the targets was evaluated by ADP-Glo or HTRF methods. Kinases are marked with dots, where the color of each kinase indicates the level of inhibition achieved by Zharp1-163. **D** Predicted binding conformation of Zharp1-163. **E** Schematic representation of the interaction patterns between Zharp1-163 and the key residues in the binding pocket of the RIPK1 kinase. **F** Indicated concentrations of Zharp1-163 did not affect RIPK3 dimerization-induced necroptosis in NIH3T3-RIPK3 cells. NIH3T3-RIPK3 cells were pretreated with the indicated concentrations of Zharp1-163 for 2 h and then treated with AP20187 (100 nM) for 24 h. Cell viability was determined by measuring ATP levels. **G** Indicated concentrations of Zharp1-163 did not affect MLKL dimerization-induced necroptosis in HeLa-MLKL (1–190) cells. HeLa-MLKL (1–190) cells were pretreated with the indicated concentrations of Zharp1-163 for 2 h and subsequently treated with AP20187 (100 nM) for 24 h. Cell viability was determined by measuring ATP levels. **H** Viability of IFNβ-primed MEFs at 18 h after stimulation with poly(I:C) plus z-VAD and treatment with Zharp1-163 (10 μM). **I** Effects of different concentrations of Zharp1-163 on WT or RIPK1 KO HT-29 cells. Cell viability was determined by measuring ATP levels. The data are presented as the mean ± SD of triplicate samples.

Previous studies have demonstrated that Zharp1-163 inhibits TNF-induced necroptosis by suppressing the activation of RIPK1 and RIPK3. We next sought to determine the binding affinity of Zharp1-163 for RIPK1 or RIPK3 via a binding assay (Fig. 4A). Consistently, Zharp1-163 strongly inhibited RIPK1 kinase activity, with an IC_50_ of approximately 406.1 nM in the ADP-Glo kinase assay (Fig. 4B). These results indicate that Zharp1-163 inhibits RIPK1 by targeting its kinase activity. Further, we evaluated the selectivity of Zharp1-163 against a panel of 81 kinases (at 1000 nM). Zharp1-163 was found to be a reasonably selective RIPK1 inhibitor, displaying >30% inhibition of three kinases (TRKB, PIK3CA and CLK1) among all of the other tested kinases (Fig. 4C and supplemental Table 1). To elucidate the binding interactions between Zharp1-163 and RIPK1 kinase, we conducted molecular docking studies using the RIPK1 crystal structure (PDB ID: 4NEU) [41]. The predicted binding conformations and interaction patterns between Zharp1-163 and the RIPK1 kinase domain are presented in Fig. 4D, E. Like the cocrystallized ligand in the 4NEU crystal complex, Zharp1-163 was predicted to act as a typical type II kinase inhibitor, interacting with the DLG (Asp156-Leu157-Gly158)-out conformation of RIPK1 (Fig. 4D, E). The 2-aminoimidazo [1,2-a] pyridine moiety of Zharp1-163 formed hydrogen bonds with Met95 in the hinge region. Furthermore, the carboxylate group of Zharp1-163 established hydrogen bonds with Asp156 in the DLG-out region. Additionally, the saturated ring of Zharp1-163 was deeply embedded within the hydrophobic allosteric pocket formed by the residues Met66, Met67, Leu70, Val76, Leu129, and Leu159, which is created by the DLG-out conformation in RIPK1 (Fig. 4D, E).

Previous studies have demonstrated that enforced dimerization or polymerization of RIPK3 or MLKL triggers necroptosis by bypassing upstream signaling [26, 42]. We further evaluated the effect of Zharp1-163 on necroptosis induced by RIPK3 dimerization or MLKL polymerization. We treated NIH3T3 cells stably expressing mouse RIPK3 fused to an FK506-binding protein (FKBP) with AP20187 to induce RIPK3 dimerization, thereby triggering necroptosis (Fig. 4F). We found that Zharp1-163 did not block RIPK3 dimerization-induced necroptosis, indicating that it had no effect on RIPK3 (Fig. 4F). HeLa cells expressing MLKL (1–190 aa) fused to DmrB can undergo necroptosis induced by MLKL polymerization upon treatment with AP20187 (Fig. 4G). Treatment with Zharp1-163 did not affect MLKL-induced necroptosis (Fig. 4G). Moreover, using RIPK1-deficient MEFs, we examined the effect of Zharp1-163 on TLR3-induced RIPK1-independent necroptosis [43] and found that Zharp1-163 did not affect cell death (Fig. 4H). This further confirms that Zharp1-163 specifically inhibits TNF-induced necroptosis in a RIPK1-dependent manner. To evaluate the role of RIPK1 in ferroptosis, we generated RIPK1-deficient HT-29 cells and found deletion of RIPK1 did not affect RSL3-induced ferroptosis (Fig. 4I). Furthermore, the ferroptosis observed in RIPK1-deficient HT-29 cells was completely suppressed by Zharp1-163, ruling out any involvement of RIPK1 in the Zharp1-163-mediated inhibition of ferroptosis (Fig. 4I). Taken together, these results demonstrate that Zharp1-163 blocks necroptosis via selective inhibition of RIPK1 kinase activity.

### Zharp1-163 ameliorates TNF-α-induced systemic inflammatory response syndrome

**Fig. 5:**
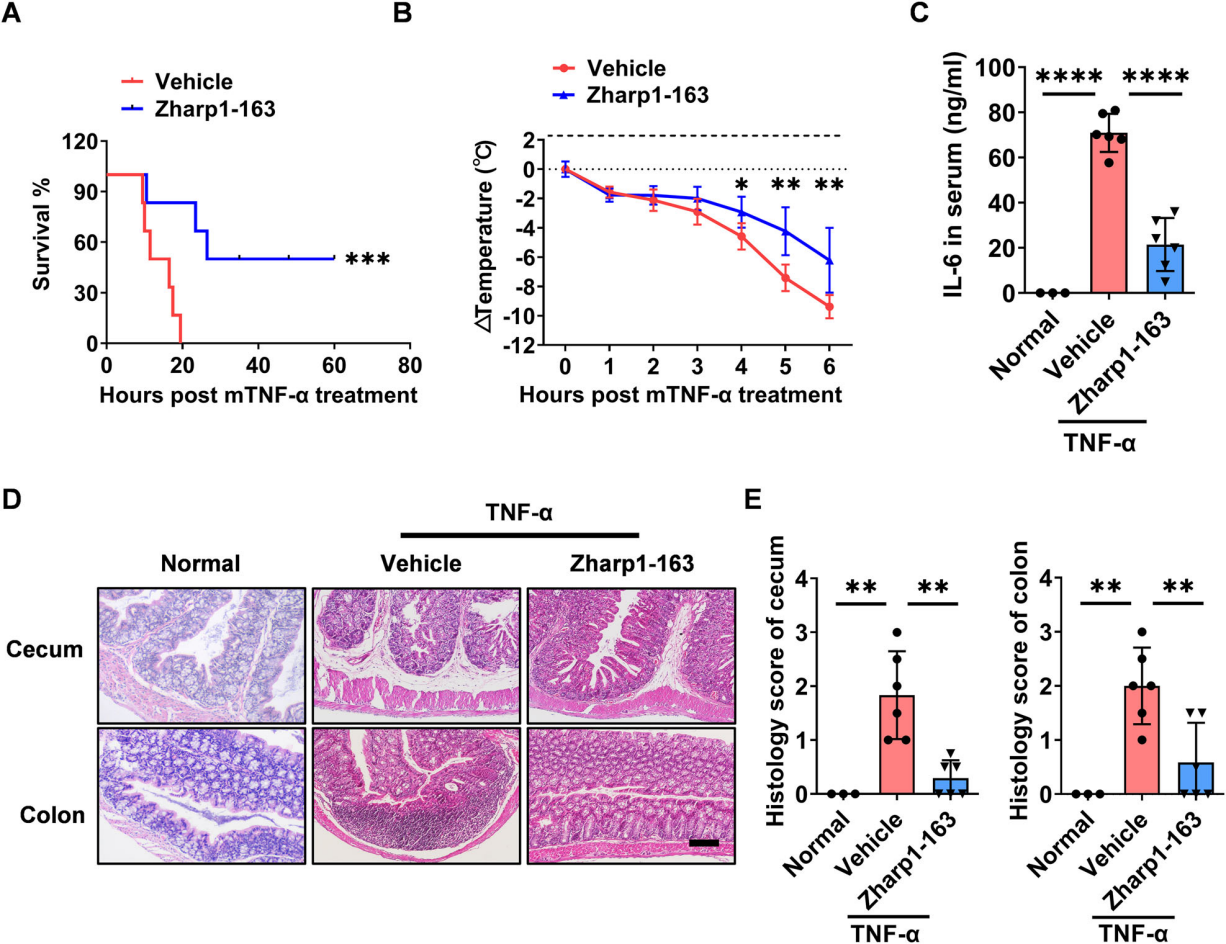
Zharp1-163 ameliorates TNF-α-induced systemic inflammatory response syndrome. **A**, **B** C57BL/6 mice were pretreated with an intraperitoneal injection of vehicle or Zharp1-163 (5 mg/kg) for 30 min before receiving an intravenous injection of TNF-α (0.2 µg/g) (vehicle, Zharp1-163 group, *n* = 6). The survival rate (**A**) and body temperature loss (**B**) were monitored. **C** The mice were sacrificed 6 h after TNF-α injection, and the level of IL-6 in the mouse serum was measured via ELISA. **D** H&E staining of the cecum and colon in the mice was performed. Scale bar, 200 μm. **E** Pathological damage to the cecum and colon was quantified. The data are presented as the mean ± SD. *P < 0.05; **P < 0.01; ***P < 0.001; ****P < 0.0001.

Given the strong biochemical and cellular activity of Zharp1-163 as a RIPK1 kinase, we aimed to evaluate its therapeutic potential in a mouse model of TNF-induced SIRS [44, 45]. C57BL/6 mice were treated with vehicle or Zharp1-163 (5 mg/kg, i.p.) for 30 min, followed by injection of TNF (0.2 µg/g). Consistently, Zharp1-163 significantly protected mice from TNF-α-induced lethality and reduced TNF-α-induced temperature loss in these mice (Fig. 5A, B). In TNF-α-induced SIRS, Zharp1-163 treatment significantly ameliorated the production of proinflammatory cytokines, including IL-6 (Fig. 5C). Moreover, the TNF-α-induced damage to the cecum and colon was attenuated by treatment with Zharp1-163 (Fig. 5D, E). These results demonstrate that the inhibition of RIPK1 by Zharp1-163 offers significant protection against TNF-induced SIRS, highlighting Zharp1-163 as a promising RIPK1 inhibitor for the development of potential anti-inflammatory therapeutics.

### Zharp1-163 alleviates kidney injury in mice

**Fig. 6:**
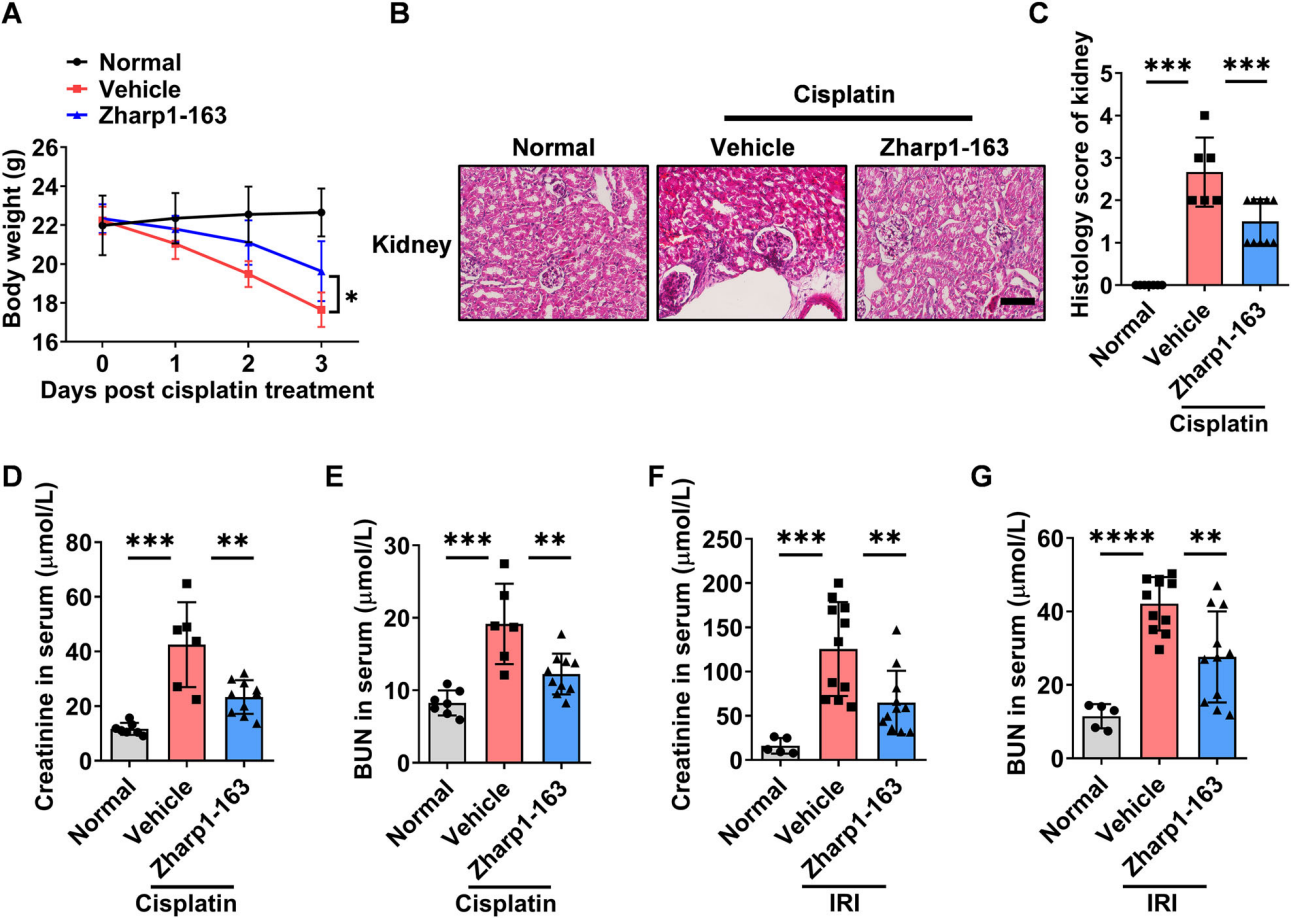
Zharp1-163 alleviates kidney injury in mice. **A–E** C57BL/6 mice were intraperitoneally injected with either vehicle or Zharp1-163 (5 mg/kg) for 30 min, followed by an intraperitoneal injection of cisplatin (20 mg/kg) (normal group, *n* = 7; vehicle group, *n* = 6; Zharp1-163 group, *n* = 10). The body weight (**A**) was monitored for three days. After the experiment, hematoxylin and eosin (H&E) staining (**B**) of the renal tissue of the mice was performed. Scale bar, 200 μm. **C** Pathological damage to the kidney was quantified. The levels of creatinine (**D**) and BUN (**E**) in mouse serum were measured. **F**, **G**. C57BL/6 mice pretreated with vehicle or Zharp1-163 (5 mg/kg) were used to establish a renal I/R injury model (normal group, *n* = 5; vehicle, Zharp1-163 group, *n* = 11). The levels of creatinine (**F**) and BUN (**G**) in mouse serum were measured. The data are presented as the mean ± SD. *P < 0.05; **P < 0.01; ***P < 0.001; ****P < 0.0001.

Necroptosis and ferroptosis have been implicated in the pathogenesis of kidney injury through their regulation of cell death and inflammation [29, 30]. Therefore, we evaluated the effect of Zharp1-163 in acute kidney disease models induced by cisplatin and ischemia-reperfusion. Cisplatin was used to induce nephrotoxicity. We observed that Zharp1-163 significantly inhibited cisplatin-induced body weight loss (Fig. 6A). H&E staining of kidney tissue revealed that cisplatin treatment resulted in mild inflammation and tubular epithelial cell damage in control mice, whereas Zharp1-163 treatment mitigated these effects (Fig. 6B, C). Consistent with its protective effect, the Zharp1-163 treatment decreased the serum creatinine and blood urea nitrogen (BUN) levels in the context of cisplatin-induced kidney injury (Fig. 6D, E). In the second model, C57BL/6 mice were treated with vehicle or Zharp1-163, followed by renal ischemia-reperfusion injury. Zharp1-163 significantly inhibited the levels of creatinine and BUN in mouse serum (Fig. 6F, G). These findings indicate that Zharp1-163 significantly alleviates acute kidney injury associated with both necroptosis and ferroptosis in models induced by cisplatin treatment and ischemia-reperfusion.

## Discussion

Studies have demonstrated that ferroptosis and necroptosis can occur simultaneously, both contributing to the progression of a single disease, such as kidney disease, heart failure, or cerebral stroke [29–32]. Therefore, the development of dual-targeting therapeutic strategies aimed at inhibiting both ferroptosis and necroptosis offers considerable therapeutic potential. In the present study, we discovered that Zharp1-163 effectively blocks both ferroptosis and necroptotic cell death, demonstrating its potential as a therapeutic agent for the treatment of acute kidney injury.

In our study, Zharp1-163 was identified as an inhibitor of lipid ROS peroxidation associated with ferroptosis, as well as an inhibitor of RIPK1 in necroptosis. RIPK1 is a member of the RIP kinase family, which includes seven members: RIPK1-RIPK7. RIPK1 regulates necroptosis through its kinase function [21]. Studies have demonstrated that deletion of RIPK1 in mice results in postnatal lethality, whereas mice expressing the kinase-dead mutant of RIPK1 survive and undergo normal development [45, 46]. Targeting RIPK1 is an effective strategy for inhibiting necroptosis and has emerged as a promising therapeutic approach for necroptosis-associated diseases. The assessment of the predicted binding mode indicated that Zharp1-163 is bound to the ATP domain in a DLG-out configuration, suggesting that it functions as a type II kinase inhibitor. This result supports the idea that Zharp1-163 specifically inhibits the kinase activity of RIPK1 without affecting other members of the RIP family. Although Zharp1-163 acts as a dual inhibitor of ferroptosis and necroptosis, our findings exclude RIPK1 as a regulatory component in RSL3-induced ferroptosis by using RIPK1-deficient cells (Fig. 4G). Future investigations into its specific target involved in ferroptosis may provide a better understanding of the molecular mechanisms underlying ferroptosis.

On the basis of the above findings, we identified Zharp1-163 as a potent dual inhibitor of ferroptosis and necroptosis in human and mouse cells. Next, we evaluated the in vivo efficacy of Zharp1-163. Zharp1-163 is a selective RIPK1 kinase inhibitor that protects against TNF-induced lethal shock, tissue damage, and the induction of inflammatory cytokines. Importantly, Zharp1-163, a dual inhibitor of ferroptosis and necroptosis, protects against loss of body weight, kidney injury, and the induction of creatinine and BUN induced by cisplatin treatment and ischemia-reperfusion. These findings highlight the critical role of Zharp1-163 in the therapeutic targeting of diseases associated with ferroptosis and necroptosis. Therefore, elucidating the precise role of Zharp1-163 in relevant disease models is essential for advancing the development of effective therapeutic interventions.

## Materials and methods

### Cells

HT-29 cells, MEFs, L929 cells, HT-1080 cells and THP-1 cells were purchased from ATCC (USA). NIH3T3-RIPK3 and HT-29-RIPK3 cells were generously provided by Dr. Xiaodong Wang (National Institute of Biological Sciences (NIBS), Beijing). The HeLa-MLKL (1–190) cell line was kindly provided by Dr. Zhigao Wang (University of Texas Southwestern Medical Center at Dallas). These cells were cultured in Dulbecco’s Modified Eagle’s Medium (HyClone) supplemented with 10% fetal bovine serum (Invitrogen) and 2 mM L-glutamine (Invitrogen).

### Drug treatments and reagents

The Smac mimetic compound was generously provided by Dr. Xiaodong Wang (National Institute of Biological Sciences, Beijing). z-VAD was purchased from Bachem. Recombinant mouse TNF-α was purchased from GenScript. Recombinant RIPK1 was purchased from SignalChem. DFO (Sigma-Aldrich, D9533) was dissolved in DMSO according to the manufacturer’s instructions. FerroOrange was purchased from DOJINDO. Creatinine and BUN assay kits were purchased from Nanjing Jiancheng Bioengineering Institute. Cisplatin was purchased from Alfa. LPS and nigericin were obtained from Sigma and InvivoGen, respectively.

### Antibodies

The following antibodies were used: anti-RIPK1 monoclonal antibody (BD Bioscience, 610459), anti-mouse-phospho-RIPK1 monoclonal antibody (Biolynx, BX60008), anti-human-phospho-RIPK1 monoclonal antibody (Cell Signaling, #65746), anti-human-RIPK3 monoclonal antibody (Cell Signaling, #13526), anti-human-phospho-RIPK3 monoclonal antibody (Abcam, ab209384), anti-mouse-RIPK3 monoclonal antibody (Prosci, 2283), anti-mouse-phospho-RIPK3 monoclonal antibody (Cell Signaling, #91702), anti-human-MLKL monoclonal antibody (Abcam, ab184718), anti-human-phospho-MLKL monoclonal antibody (Abcam, ab187091), anti-mouse-MLKL monoclonal antibody (Abgent, AP14272b), anti-mouse-phospho-MLKL monoclonal antibody (Abcam, ab196436), anti-GPX4 monoclonal antibody (Cell Signaling, #52455) and anti-β-actin monoclonal antibody (Sigma-Aldrich, A2066).

### Cell survival assay

Cell viability was assessed using the CellTiter-Glo® Luminescent Cell Viability Assay Kit, following the manufacturer’s instructions (Promega). Luminescence was measured with a SpectraMax i3x (Molecular Devices).

### Measurement of qPCR

Total RNA was extracted from HT-1080 cells via an RNA-Quick Purification Kit (OMEGA, USA) according to the manufacturer’s instructions. QPCR was conducted via SYBR Green Master Mix (Bimake, USA) along with the appropriate primers on the Roche LightCycler 480 II system, with the data normalized to GAPDH. The mRNA expression levels of *PTGS2* and *CHAC1* were detected. The sequences of primers used were as follows: human *PTGS2* sense: TTCCTCCTGTGCCTGATGATT; antisense: AAACTGATGCGTGAAGTGCTG; human *CHAC1* sense: GACGCTCCTTGAAGATCATGAG; antisense: CAGCAAGTATTCAAGGTTGTGG; and human GAPDH sense AATGGGCAGCCGTTAGGAAA, antisense: GCCCAATACGACCAAATCAGAG.

### Western blot analysis

The cell pellet was harvested and lysed in protein lysis buffer (20 mM Tris-HCl (pH 7.4), 150 mM NaCl, 10% glycerol, 1% Triton X-100, 1 mM Na3VO4, 25 mM β-glycerol phosphate, 0.1 mM PMSF) supplemented with a complete protease inhibitor set (Roche) for 20 min. The cell lysates were then centrifuged at 13,000 × *g* for 20 min at 4 °C. The supernatants were collected and analyzed using western blotting.

### p-RIPK1 immunofluorescence staining

HT-29 cells were seeded in six-well plates at a density of 800,000 cells per well. After overnight incubation, the cells were pretreated with Zharp1-163. Two hours later, the cells were treated with TNF-α (40 ng/ml), Smac mimetic (100 nM) and z-VAD (20 μM). Immunofluorescence staining was performed at 8 h post-treatment.

### RIPK3 puncta immunofluorescence staining

HT-29 cells expressing Flag-RIPK3 were plated in a chamber slide and allowed to culture overnight. Next, the cells were pretreated with the indicated concentrations of Zharp1-163 for 2 h before being exposed to TNF-α (40 ng/ml), Smac mimetic (100 nM) and z-VAD (20 μM) for 8 h. After treatment, the cells were washed with phosphate-buffered saline (PBS) and then fixed with 4% paraformaldehyde for 20 min. After fixation, the cells were rinsed three times with PBS and treated with a solution of 0.25% Triton X-100 in PBS for another 10 min. After permeabilization, the cells were blocked for 1 h with 5% BSA in PBS. The samples were then incubated with an anti-flag antibody at 4 °C overnight. The next day, the cells were warmed to room temperature for 30 min, washed three times with PBS, and then incubated with a secondary antibody. The nuclei were stained with DAPI. Ultimately, the stained cells were observed, and images were captured via an Olympus confocal microscope.

### In vitro kinase activity assay

The recombinant RIPK1 protein was incubated with DMSO or Zharp1-163 for 15 min in assay buffer (25 mM HEPES (pH 7.2), 20 mM MgCl2, 12.5 mM MnCl2, 12.5 mM β-glycerol phosphate, 5 mM EGTA, 2 mM EDTA, and 2 mM DTT). The reaction was then conducted at room temperature for 2 h, with the addition of ATP (50 μM) and the substrate MBP (20 μM). Luminescence was analyzed using the ADP-Glo Kinase Assay Kit according to the manufacturer’s instructions (Promega).

### DPPH radical scavenging activity

DPPH radicals were used to measure the free radical scavenging capacity of different concentrations of Zharp1-163. DPPH dissolved in ethanol was mixed with different concentrations of Zharp1-163. After incubation at room temperature in the dark for 30 min, detection and calculation were performed according to the reagent instructions (Beijing Solarbio Science & Technology Co., Ltd.).

### Measurement of ROS levels

The ROS levels in HT-1080 cells were measured by flow cytometry after pretreatment with the indicated concentrations of Zharp1-163 for 2 h, followed by induction with RSL3 for 5 h. The ROS levels were analyzed according to the manufacturer’s instructions (Beyotime).

### Detection of GSH levels

The GSH levels in HT-1080 cells were analyzed after pretreatment with the indicated concentrations of Zharp1-163 for 2 h, followed by induction with erastin (10 μM). Luminescence was measured to determine GSH levels according to the manufacturer’s instructions (Promega).

### Iron staining

HT-1080 cells were seeded in fluorescence-compatible culture dishes and incubated overnight. The cells were washed three times with serum-free medium and then incubated at room temperature in a medium containing the test compounds or deferoxamine (DFO) for 5 h. Subsequently, 1 μM FerroOrange (DOJINGO) working solution was added, and the cells were observed under a fluorescence microscope.

### ELISA

The levels of IL-6 in mouse serum were measured using ELISA kits from Invitrogen, following the manufacturer’s instructions.

### Source of the animals

Male C57BL/6 mice were purchased from Vital River Laboratory Animal Technology Co., Ltd. (Beijing, China). All mice were maintained under standard conditions and utilized at 6–8 weeks of age and weighed approximately 20 g. These mice were then housed in a specific pathogen-free environment at the Suzhou Institutes of Systems Medicine.

### Ethics approval statement

All animal experiments were performed in accordance with protocols approved by the Suzhou Institutes of Systems Medicine Institutional Animal Care and Use Committee (ISM-IACUC-0037-R).

### TNF-induced SIRS

Zharp1-163 was diluted in sterile PBS. C57BL/6 mice were randomly assigned to two groups and pretreated with either vehicle or Zharp1-163 (5 mg/kg) via intraperitoneal injection approximately 30 min prior to challenge. Subsequently, the mice were administered mouse TNF-α (0.2 μg/g) via intravenous tail injection. Body temperature loss and mortality in the mice were continuously monitored following TNF-α administration. Mouse serum was collected 6 h after TNF-α injection.

### Histopathological analysis

After TNF-α-induced systemic inflammatory response syndrome, the cecum and colon were preserved in 4% paraformaldehyde for 2 days, sliced into sections, and subsequently stained with hematoxylin and eosin (H&E). The degree of cecal damage and injury of the colon was assessed by a method described previously [47]. Images were captured via an Olympus CX33 with a QT-200 USB 3.0 camera.

### Ischemia-reperfusion-induced kidney injury

C57BL/6 mice were purchased from Beijing Vital River Laboratory Animal Technology Co., Ltd. The mice were randomly divided into three groups: the sham-operated normal group (Normal), the vehicle group and the Zharp1-163 group (5 mg/kg). The left renal artery of mice pretreated with either vehicle or Zharp1-163 (5 mg/kg) for 2 h was isolated and clamped for 45 min via a nontraumatic artery clamp following right nephrectomy. Reperfusion was subsequently performed. The mice were sacrificed after 24 h. The normal group was subjected to the same process without ligation of the artery. The levels of creatinine and BUN in mouse serum were analyzed according to the manufacturer’s instructions (Nanjing Jiancheng Bioengineering Institute).

### Cisplatin-induced acute kidney injury

C57BL/6 mice were purchased from Beijing Vital River Laboratory Animal Technology Co., Ltd. The mice were randomly divided into three groups: the sham-operated control group (Normal), the vehicle group and the Zharp1-163 group (5 mg/kg). The mice were intraperitoneally injected with either vehicle or Zharp1-163 (5 mg/kg) for 30 min, followed by an intraperitoneal injection of cisplatin (20 mg/kg). The body weight was monitored for three days. Kidney tissues were collected for H&E staining. Moreover, creatinine and BUN in mouse serum were collected and analyzed according to the manufacturer’s instructions. (Nanjing Jiancheng Bioengineering Institute). Similarly, serum creatinine and BUN levels were collected and analyzed following the cisplatin-induced acute kidney injury model. The degree of kidney injury was assessed by a method described previously [48]. Images were captured via an Olympus CX33 with a QT-200 USB 3.0 camera.

### Statistical analysis

The numerical data are presented as the mean ± SD. All experiments were repeated at least 3 times with similar results. The significance of differences was evaluated via *t*-tests via GraphPad Prism software. The significance levels are as follows: n.s., not significant; *P < 0.05; **P < 0.01; ***P < 0.001; ****P < 0.0001.

## Supplementary information

Supplemental Information-Supplemental figure 1 and table 1

Supplemental Material-Zharp1-163-Original Western Blots

## Data Availability

All the data and experimental details in this article may be obtained from the corresponding author upon reasonable request.
