# Supplementary material for: Discovery of Zharp1-163 as a dual inhibitor of ferroptosis and necroptosis for the treatment of inflammatory disorders and kidney injury
Source: Cell Death Discov. 2025 Aug 28;11:413. doi: 10.1038/s41420-025-02693-5 (PMC12394658; doi:10.1038/s41420-025-02693-5)
Supplement: Supplementary file 1 — Supplemental Information-Supplemental figure 1 and table 1 [file 41420_2025_2693_MOESM1_ESM.docx]

**Discovery of Zharp1-163 as a dual inhibitor of ferroptosis and necroptosis for the treatment of inflammatory disorders and kidney injury**

Yuting Ji^1,2#^, Shujing Du^1#^, Jingjing Li^1#^, Haikuo Ma^3#^, Xinhui Wang^1^, Yongjin Hao^3^, Zhanhui Li^3^, Haohao Lu^1^, Hao Liu^4^, Chengkui Yang^1^*, Xiaohu Zhang^3^* and Sudan He^1^*

^1^State Key Laboratory of Common Mechanism Research for Major Diseases, and Key Laboratory of Synthetic Biology Regulatory Elements, Suzhou Institute of Systems Medicine, Chinese Academy of Medical Sciences & Peking Union Medical College, Suzhou 215123, Jiangsu, China;

^2^School of Life Science and Technology, China Pharmaceutical University, Nanjing, Jiangsu, China;

^3^Jiangsu Key Laboratory of Neuropsychiatric Diseases and College of Pharmaceutical Sciences, Soochow University, Suzhou 215123, China;

^4^School of Chemistry, Chemical Engineering and Life Science, Wuhan University of Technology, Wuhan 430070, Hubei, People's Republic of china.

^#^These authors equally contributed to this study.

* Corresponding authors:

Dr.Chengkui Yang (Email: yangck222@163.com;), Dr. Xiaohu Zhang (Email: [xiaohuzhang@suda.edu.cn](mailto:xiaohuzhang@suda.edu.cn)), or Dr. Sudan He (Email: [hesd@ism.pumc.edu.cn](mailto:hesd@ism.pumc.edu.cn))

**Detailed Synthetic Procedures**

General reaction progress was monitored by analytical thin layer chromatography performed on silica gel HSGF254 pre-coated plates. Organic solutions were dried over anhydrous Na_2_SO_4_, and the solvents were removed under reduced pressure. Final compounds were purified with silica gel 100-200 mesh for column chromatography. ^1^H NMR and ^13^C NMR were obtained on 300 MHz (Varian), 400 MHz (Varian) or 600 MHz (Varian) spectrometers. Chemical shifts were given in ppm using tetramethylsilane as internal standard. Data for ^1^H NMR are reported as follows: chemical shift, multiplicity (s = singlet, d = doublet, t = triplet, q = quartet, m = multiplet, br = broad), coupling constants and integration. Mass spectra were obtained using an Agilent 1100 LC/MSD Trap SL version Mass Spectrometer. HRMS analysis was recorded on an Agilent 6540 UHD Accurate-Mass QTOF LC/MS.


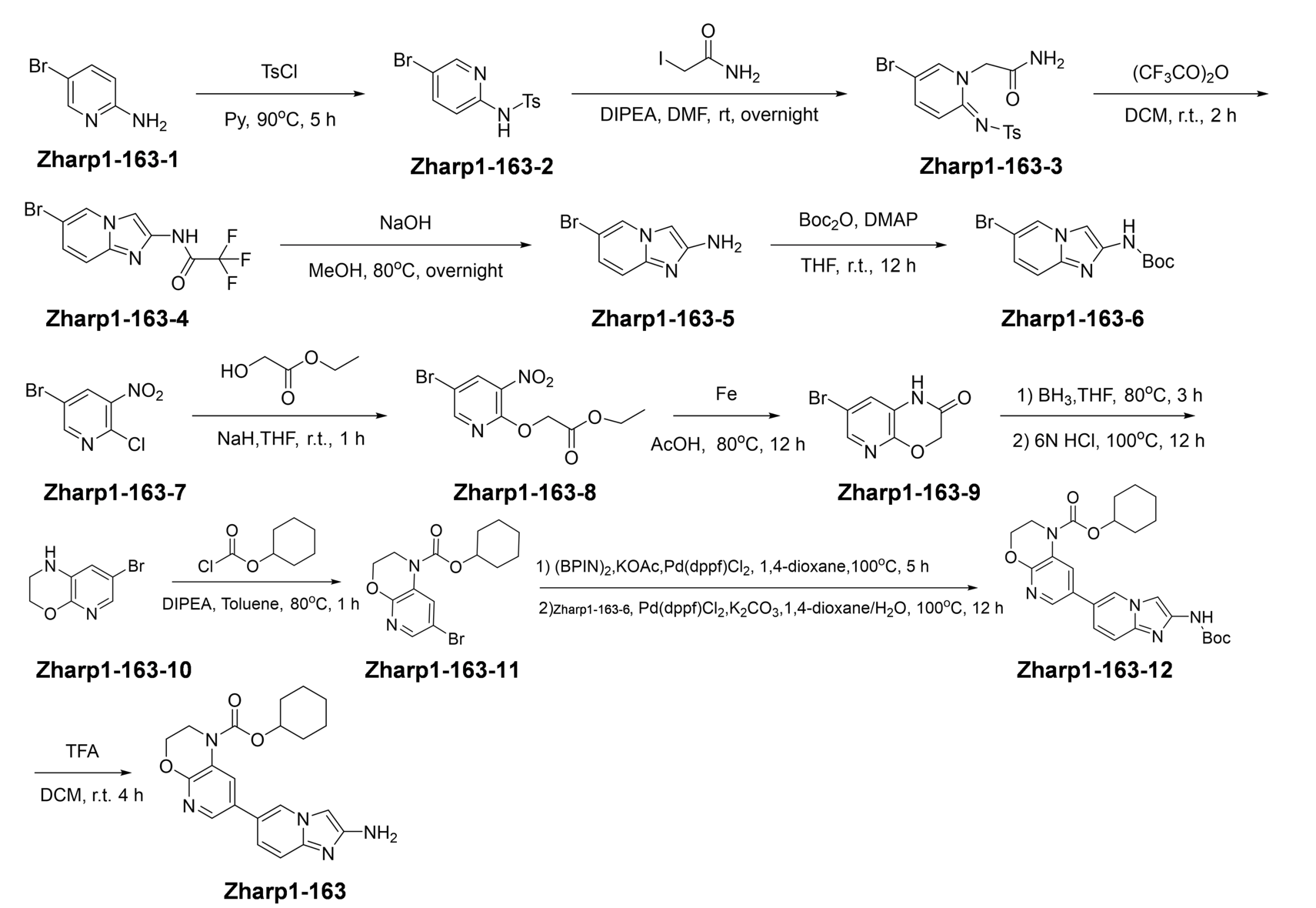


**Supplemental Figure 1**: Synthetic scheme for **Zharp1-163.**

1. Synthesis of **Zharp1-163**

**Zharp1-163-2**: N-(5-bromopyridin-2-yl)-4-methylbenzenesulfonamide

To a solution of **Zharp1-163-1** (308 g, 1720 mmol) in dry pyridine (1.4 L) was added TsCl (366 g, 1900 mmol) at room temperature. After the mixture was stirred at 90℃ for 5 h, the mixture was quenched with water (2.5 L). The resulting precipitate was filtered. The cake was washed with water and dried to give a yellow solid (587 g, crude). ^1^H NMR (400 MHz, DMSO-*d_6_*) δ 11.24 (br s, 1H), 8.27 (s, 1H), 7.89 (d, *J* = 8.0 Hz, 1H), 7.78 (d, *J* = 8.0 Hz, 2H), 7.36-7.32 (m, 2H), 7.02 (d, *J* = 8.8 Hz, 1H), 2.35 (s, 3H).

**Zharp1-163-3**: (Z)-2-(5-bromo-2-(tosylimino)pyridin-1(2H)-yl)acetamide

To a solution of **Zharp1-163-2** (587 g, 1800 mmol) in dry DMF (1.8 L) was added 2-iodoacetamide (383 g, 2070 mmol), DIPEA (278 g, 2160 mmol). The mixture was stirred at room temperature overnight. The reaction was added water (20 L) and the resulting precipitate was filtered, dried to give a white solid (590 g, 90%). ^1^H NMR (400 MHz, DMSO-*d_6_*) δ 8.37 (s, 1H), 7.88 (d, *J* = 9.6 Hz, 1H), 7.79 (s, 1H), 7.65 (d, *J* = 7.6 Hz, 2H), 7.40 (s, 1H), 7.33 – 7.23 (m, 3H), 4.78 (s, 2H), 2.34 (s, 3H).

**Zharp1-163-4**: N-(6-bromoimidazo[1,2-a]pyridin-2-yl)-2,2,2-trifluoroacetamide

To a solution of **Zharp1-163-3** (150 g, 391 mmol) in dry DCM (1.5 L) was added trifluoroacetic anhydride (164 g, 783 mmol) at 0℃. After carried out at room temperature for 2 h, the mixture was added ice water (3 L) and adjusted by sat. aq. NaHCO_3_ to pH = 7. The resulting precipitate was filtered, dried to give a white solid (120 g, 50%). ^1^H NMR (400 MHz, DMSO-*d_6_*) δ 12.55 (br s, 1H), 8.96 (s, 1H), 8.24 (s, 1H), 7.51 (d, *J* = 8.8 Hz, 1H), 7.41 (d, *J* = 8.8 Hz, 1H).

**Zharp1-163-5**: 6-bromoimidazo[1,2-a]pyridin-2-amine

To a solution of **Zharp1-163-4** (110 g, 357 mmol) in MeOH (1.0 L) was added a solution of NaOH (17 g, 428 mmol) in water (420 mL). The mixture was stirred at 80℃ overnight. The reaction was added saturated aqueous NaCl (1.0 L) and extracted with DCM (800 mL*3). The organic layers were combined and then dried over Na_2_SO_4_, filtered and concentrated to give a yellow solid (75 g, 99%). ^1^H NMR (400 MHz, DMSO-*d_6_*) δ 8.60 (s, 1H), 7.13 (d, *J* = 9.2 Hz, 1H), 7.07 (d, *J* = 9.2 Hz, 1H), 7.00 (s, 1H), 5.19 (br s, 2H).

**Zharp1-163-6**: tert-butyl (6-bromoimidazo[1,2-a]pyridin-2-yl)carbamate

To a solution of **Zharp1-163-5** (1.0 g, 4.7 mmol) in dry DCM (20 mL) was added DMAP (57 mg, 0.47 mmol), Boc_2_O (1.6 g, 7.1 mmol). The mixture was stirred at room temperature for 12 h and then concentrated. The residue was purified by silica gel column chromatography (petroleum ether/ethyl acetate = 1/1) to give the title compound as a white solid (1.5 g, 100%).^1^H NMR (400 MHz, DMSO-*d_6_*) δ 10.02 (s, 1H), 8.82 (s, 1H), 7.85 (s, 1H), 7.36 (d, *J* = 9.2 Hz, 1H), 7.27 (d, *J* = 9.2 Hz, 1H), 1.47 (s, 9H).

**Zharp1-163-8**: ethyl 2-((5-bromo-3-nitropyridin-2-yl)oxy)acetate

To a solution of **Zharp1-163-7** (1.0 g, 4.2 mmol) and ethyl 2-hydroxyacetate (484 mg, 4.7 mmol) in dry THF (50 mL) was added 60% NaH (484 mg, 4.7 mmol) at 0℃. After carried out at room temperature for 1 h, the mixture was added water (30 mL) and extracted with EA (30 mL*3). The organic layers were combined and then dried over Na_2_SO_4_, filtered and concentrated. The residue was purified by silica gel column chromatography (petroleum ether/ethyl acetate = 1/1) to give the title compound as a yellow solid (1.1 g, 89%). ^1^H NMR (400 MHz, CDCl_3_) δ 8.46 (d, *J* = 2.0 Hz, 1H), 8.39 (d, *J* = 1.6 Hz, 1H), 5.05 (s, 2H), 4.26-4.17 (m, 2H), 1.28 (t, *J* = 7.2 Hz, 3H).

**Zharp1-163-9**: 7-bromo-1H-pyrido[2,3-b][1,4]oxazin-2(3H)-one

To a solution of **Zharp1-163-8** (1.1 g, 3.7 mmol) in AcOH (23 mL) was added Fe powder (5.5 g, 98 mmol) at 80℃. After stirred for 12 h at 80℃, the mixture was filtered and the filtrate was concentrated. The residue was adjusted by sat. aq. Na_2_CO_3_ to pH = 9 and extracted with EA (20 mL*3). The organic phase was concentrated and purified by silica gel column chromatography (petroleum ether/ethyl acetate = 1/1) to give the title compound as a white solid (700 mg, 90%).^1^H NMR (400 MHz, DMSO-*d_6_*) δ 10.94 (s, 1H), 7.88 (s, 1H), 7.33 (s, 1H), 4.81 (s, 2H).

**Zharp1-163-10**: 7-bromo-2,3-dihydro-1H-pyrido[2,3-b][1,4]oxazine

To a solution of **Zharp1-163-9** (1.5 g, 6.6 mmol) in dry THF (20 mL) was added 1 N BH_3_ in THF (50 mL, 50 mmol) at 0℃. The mixture was stirred at 80℃ for 3 h. Then, the mixture was added 6 N HCl in water (50 mL, 300 mmol ) and stirred at 100℃ for 12 h. The reaction was adjusted by sat. aq. Na_2_CO_3_ to pH = 9 and extracted with EA (20 mL*3). The organic phase was concentrated and purified by silica gel column chromatography (petroleum ether/ethyl acetate = 1/1) to give the title compound as a white solid (1.0 g, 68%).^1^H NMR (400 MHz, DMSO-*d_6_*) δ 7.38 (s, 1H), 7.01 (s, 1H), 6.36 (s, 1H), 4.20-4.25 (m, 2H), 3.21-3.27 (m, 2H).

**Zharp1-163-11**: cyclohexyl 7-bromo-2,3-dihydro-1H-pyrido[2,3-b][1,4]oxazine-1-carboxylate

To a solution of **Zharp1-163-10** (2.0 g, 9.3 mmol) in toluene (50 mL) was added DIPEA (5.6 g, 37 mmol), cyclohexyl carbonochloridate (3.0 g, 18 mmol) at 0℃. The mixture was stirred at 80℃ for 1 h. The reaction was concentrated and purified by silica gel column chromatography (petroleum ether/ethyl acetate = 10/1) to give the title compound as a colorless oil (2.8 g, 93%).^1^H NMR (400 MHz, DMSO-*d_6_*) δ 8.49 (br s, 1H), 7.98 (s, 1H), 4.84-4.71 (m, 1H), 4.39 (t, *J* = 4.0 Hz, 2H), 3.86 (t, *J* = 4.0 Hz, 2H), 1.90-1.77 (m, 2H), 1.73-1.60 (m, 2H), 1.59-1.30 (m, 6H).

**Zharp1-163-12**: cyclohexyl 7-(2-((tert-butoxycarbonyl)amino)imidazo[1,2-a]pyridin-6-yl)-2,3-dihydro-1H-pyrido[2,3-b][1,4]oxazine-1-carboxylate

A 100 mL flask was charged with **Zharp1-163-11** (1.5 g, 4.4 mmol), bis(pinacolato)diboron (1.6 g, 6.3 mmol), KOAc (1.0 mg, 11 mmol) and Pd(dppf)Cl_2_ (230 mg, 0.32 mmol) followed by addition of 30 mL 1,4-dioxane. The equipment was evacuated and refilled with N_2_ three times. The reaction was carried out at 100 ℃ for 5 h. After cooled to room temperature, the mixture was added **Zharp1-163-6** (980 mg, 3.2 mmol), K_2_CO_3_ (1.1 mg, 7.8 mmol), Pd(dppf)Cl_2_ (230 mg, 0.32 mmol) and water (2.0 mL). The equipment was evacuated and refilled with N_2_ three times. The reaction was carried out at 100 ℃ for 12 h. The mixture was concentrated and purified by silica gel column chromatography (DCM/MeOH = 70/1) to give the title compound as a white solid (980 mg, 63%).^1^H NMR (400 MHz, DMSO-*d_6_*) δ 9.95 (s, 1H), 8.81 (s, 1H), 8.56 (s, 1H), 8.20 (s, 1H), 7.87 (s, 1H), 7.48 (d, *J* = 8.8 Hz, 1H), 7.41 (d, *J* = 8.8 Hz, 1H), 4.88 – 4.68 (m, 1H), 4.46 – 4.36 (m, 2H), 3.94 – 3.89 (m, 2H), 1.95 – 1.82 (m, 2H), 1.72 – 1.63 (m, 2H), 1.61 – 1.52 (m, 3H), 1.49 (s, 9H), 1.42 – 1.18 (m, 3H).

**Zharp1-163**: cyclohexyl 7-(2-aminoimidazo[1,2-a]pyridin-6-yl)-2,3-dihydro-1H-pyrido[2,3-b][1,4]oxazine-1-carboxylate

To a solution of **Zharp1-163-12** (1.7 g, 3.5 mmol) in dry DCM (20 mL) was added TFA (4.0 g, 35 mmol). The mixture was stirred at room temperature for 4 h. The reaction was adjusted by sat. aq. NaHCO_3_ to pH = 8 and extracted with DCM (20 mL*3). The organic layer was dried over Na_2_SO_4_, filtered and concentrated to give the target compound (800 mg, 58%).^1^H NMR (400 MHz, DMSO-*d_6_*) δ 8.63 (s, 1H), 8.53 (s, 1H), 8.16 (s, 1H), 7.32 – 7.20 (m, 2H), 7.03 (s, 1H), 5.12 (s, 2H), 4.85 – 4.70 (m, 1H), 4.44 – 4.37 (m, 2H), 3.94 – 3.86 (m, 2H), 1.86 – 1.83 (m, 2H), 1.71 – 1.62 (m, 2H), 1.58 – 1.46 (m, 3H), 1.40 – 1.27 (m, 3H). ^13^C NMR (151 MHz, DMSO-*d_6_*) δ 153.0, 152.7, 151.8, 141.8, 139.6, 128.4, 127.7, 122.4, 122.2, 121.5, 120.8, 113.9, 93.9, 74.7, 65.5, 42.2, 31.4, 25.3, 23.3. HRMS (ESI) calcd for C_21_H_23_N_5_O_3_ [M + H] ^+^, 394.1879; found, 394.1880.

**The Procedures of Molecular docking**

The structure preparation was carried out using the Molecular Operating Environment (MOE) v2022 package, beginning with the Structure Prepare application, followed by proton addition and optimization using the Protonate3D application. Subsequent docking simulations were conducted using the same MOE version. The ligand parameters were computed using the Extended Hückel Theory (Amber14:EHT) as implemented in MOE, and an "Induced Fit" docking (IFD) protocol was employed for all protein-ligand complexes. Within the IFD protocol, initial ligand placement was accomplished using the Triangle Matcher algorithm, with poses initially scored using the London ΔG scoring function and subsequently filtered using the GBVI/WSA ΔG score. To ensure comprehensive sampling of possible binding modes, the Triangle Matcher generated 250 initial poses for each case, and the active site was refined using the Amber14:EHT force field implemented in MOE.


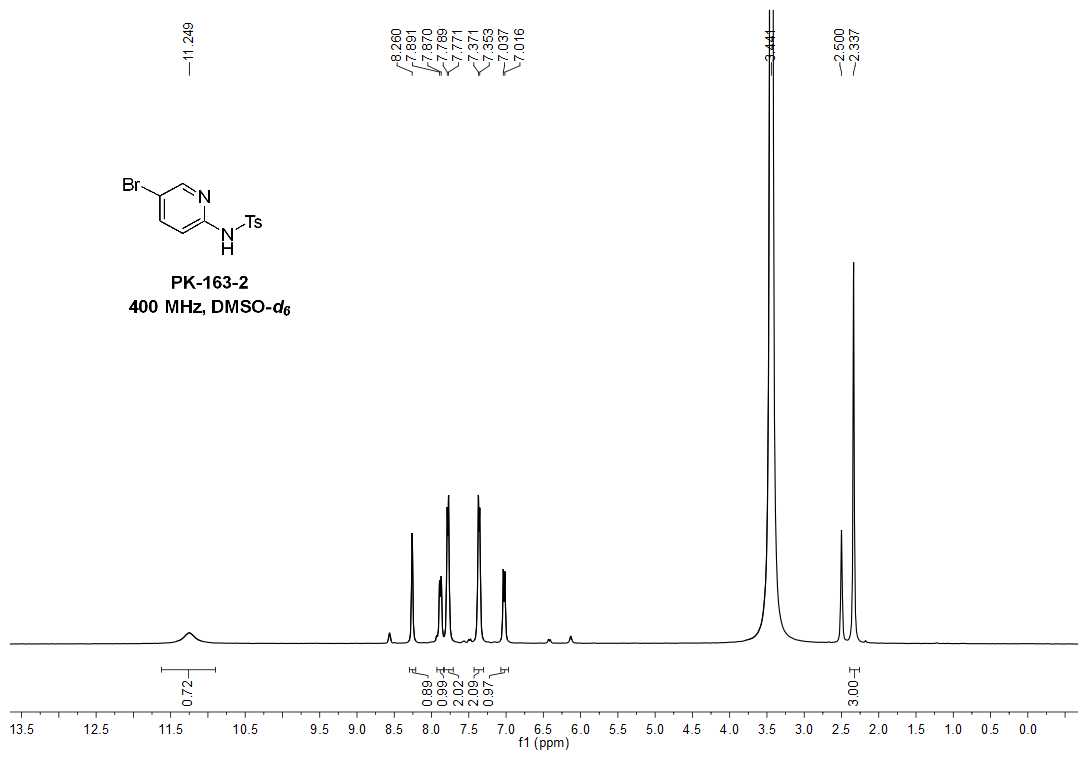


**Supplemental Figure 1**. ^1^H NMR spectra for **Zharp1-163-2**


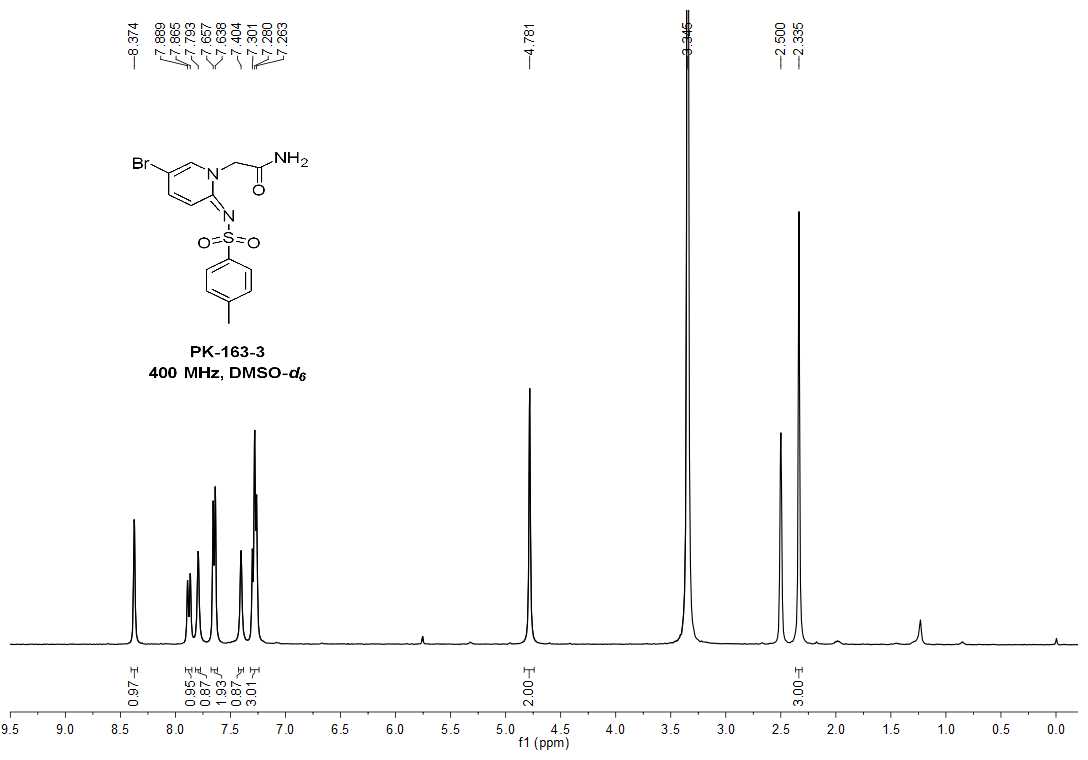


**Supplemental Figure 2**. ^1^H NMR spectra for **Zharp1-163-3**


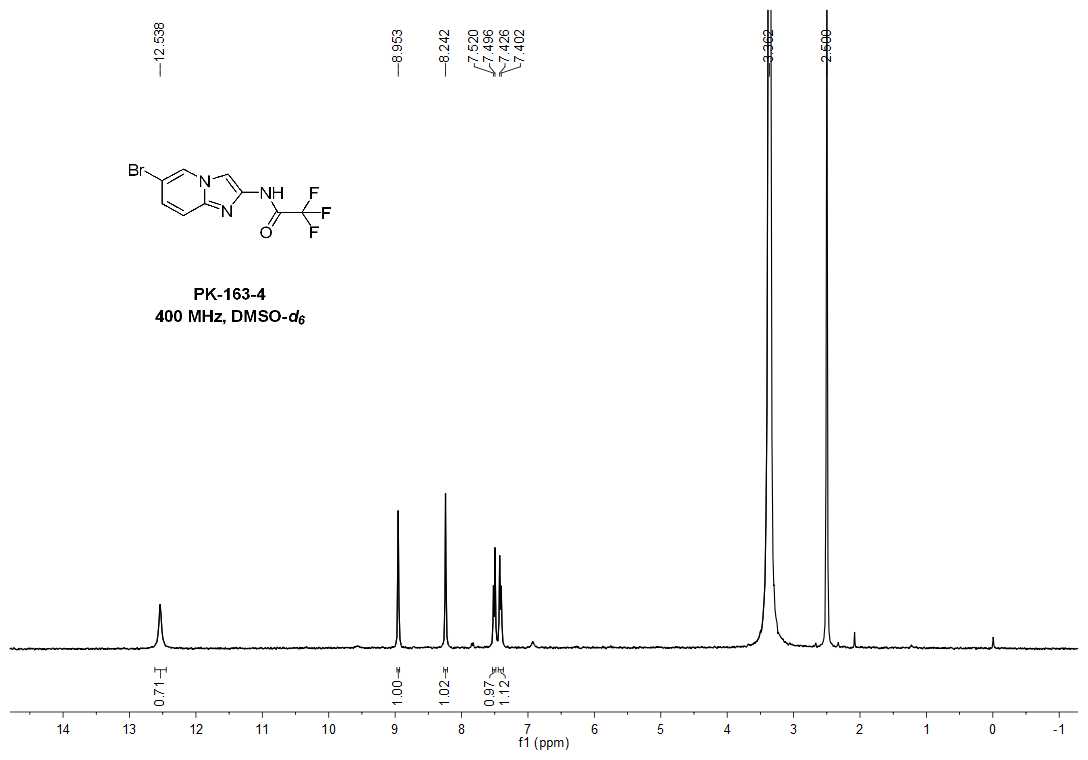


**Supplemental Figure 3**. ^1^H NMR spectra for **Zharp1-163-4**


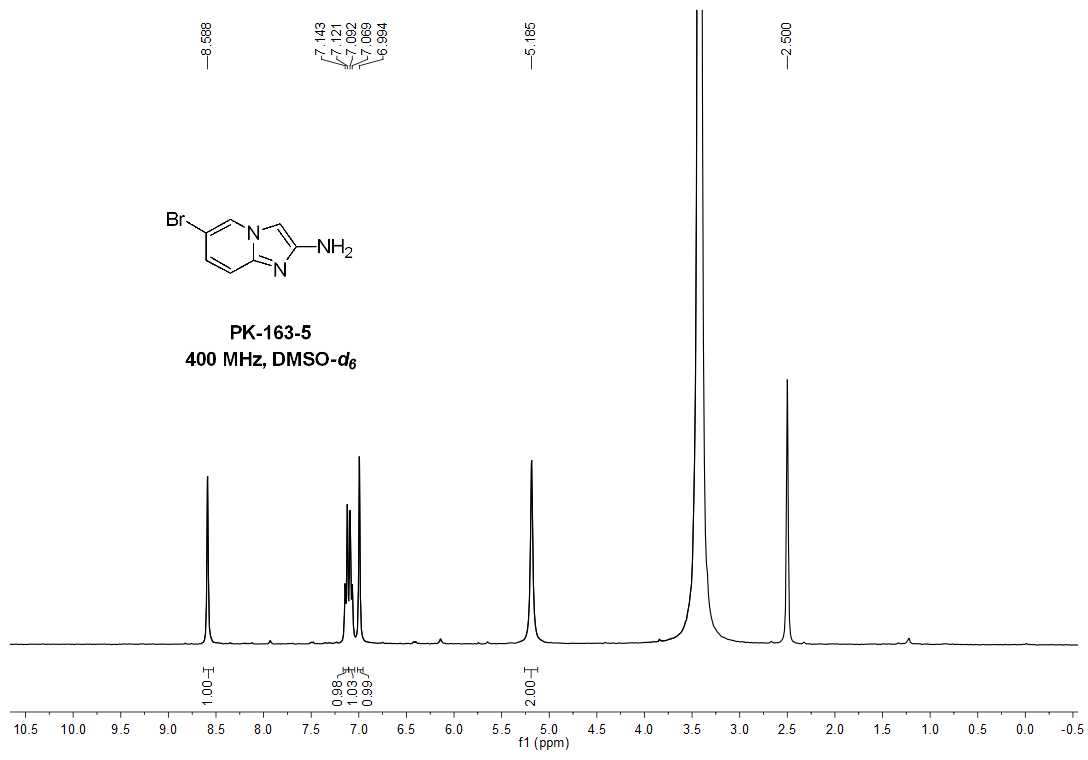


**Supplemental Figure 4**. ^1^H NMR spectra for **Zharp1-163-5**


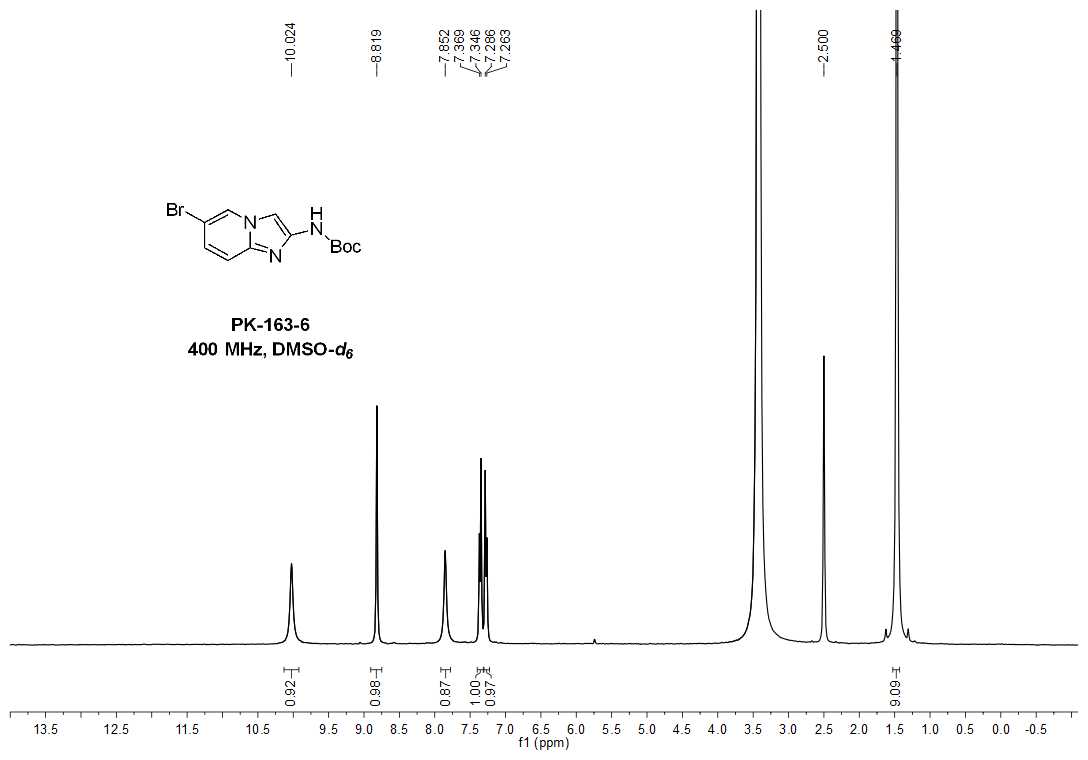


**Supplemental Figure 5**. ^1^H NMR spectra for **Zharp1-163-6**


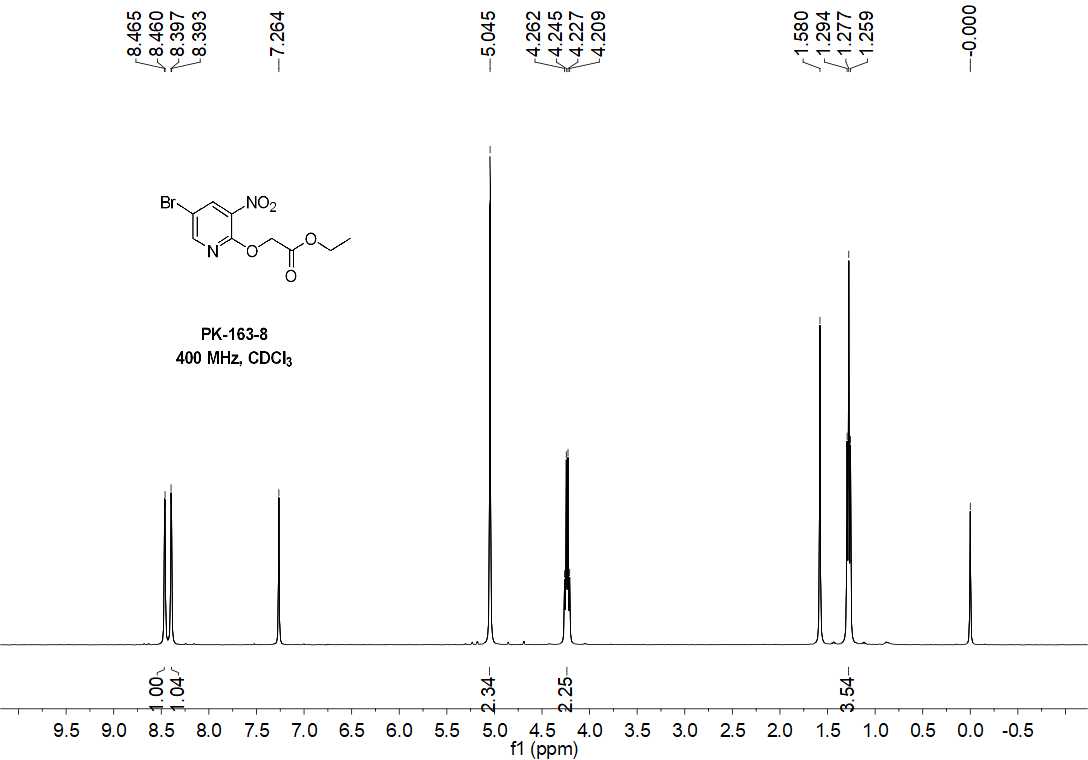


**Supplemental Figure 6**. ^1^H NMR spectra for **Zharp1-163-8**


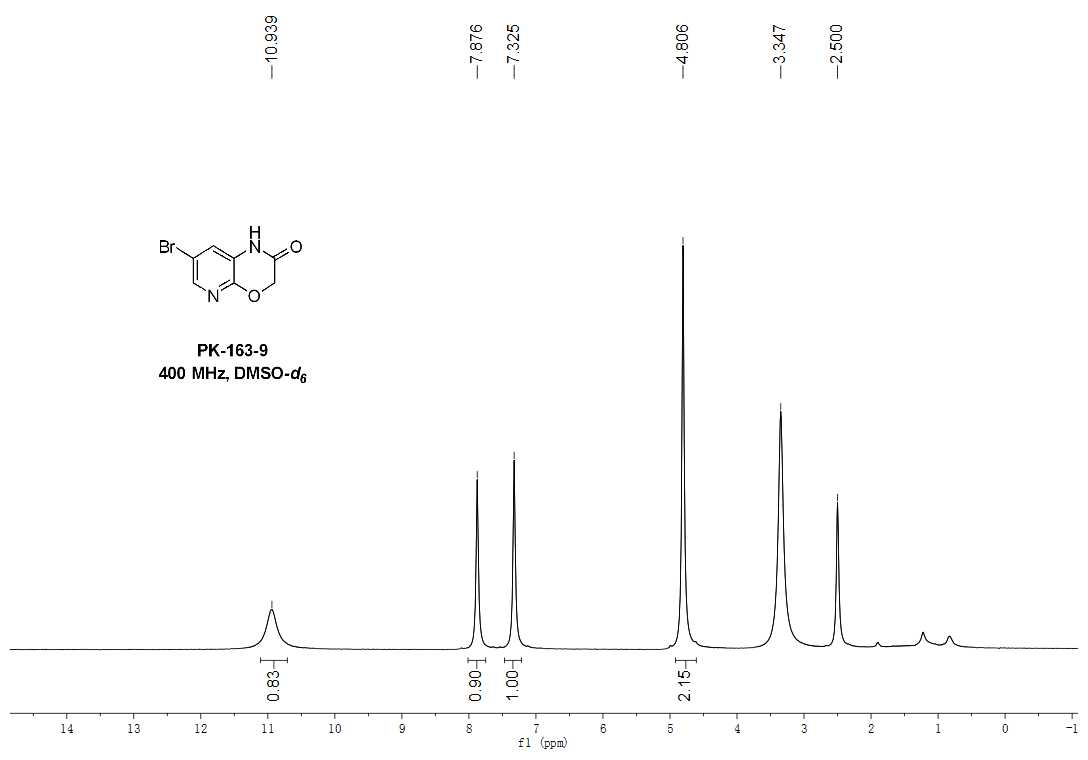


**Supplemental Figure 7**. ^1^H NMR spectra for **Zharp1-163-9**


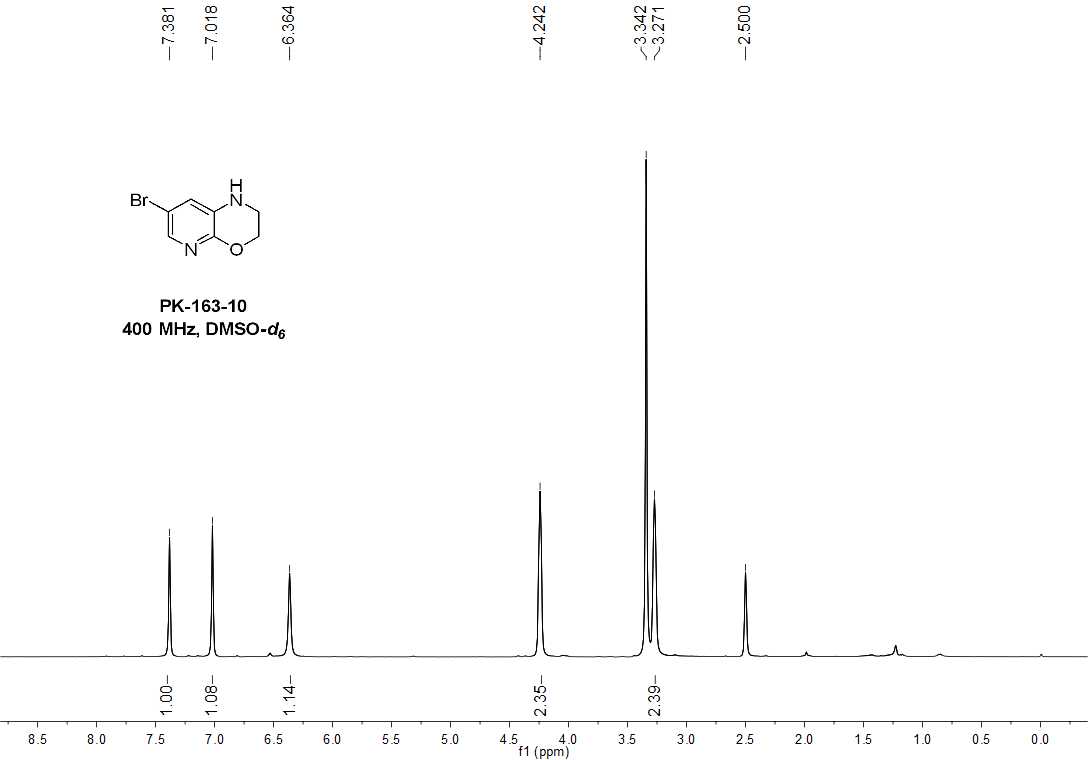


**Supplemental Figure 8**. ^1^H NMR spectra for **Zharp1-163-10**


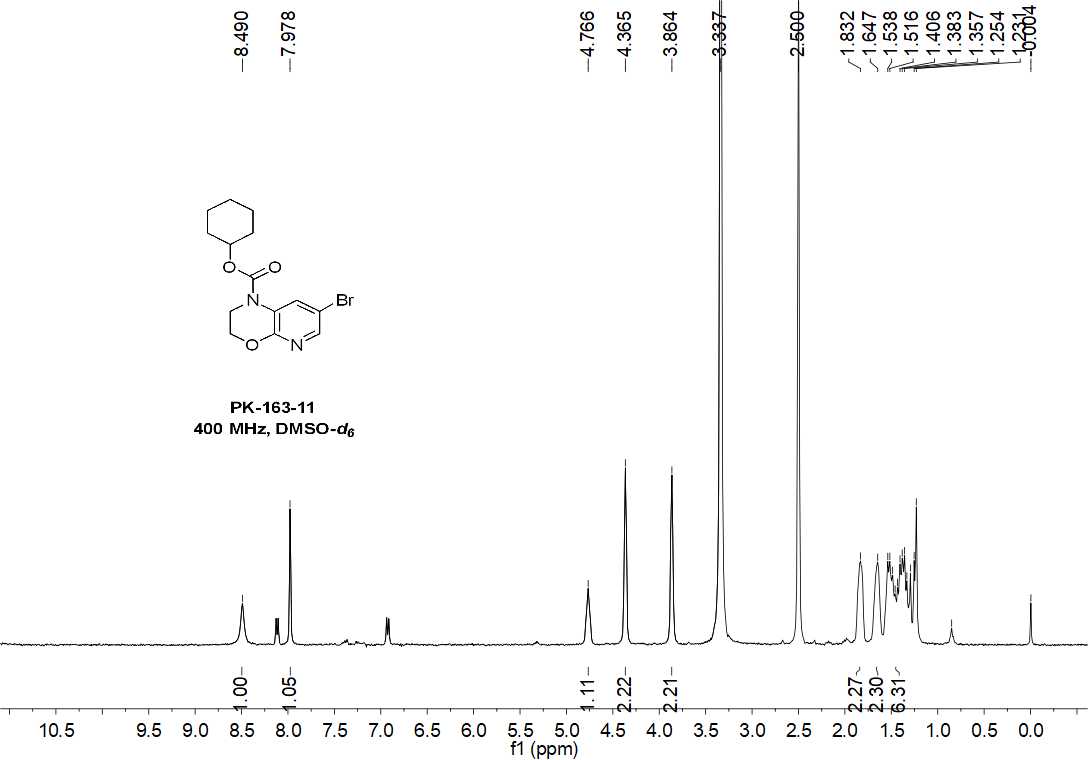


**Supplemental Figure 9**. ^1^H NMR spectra for **Zharp1-163-11**


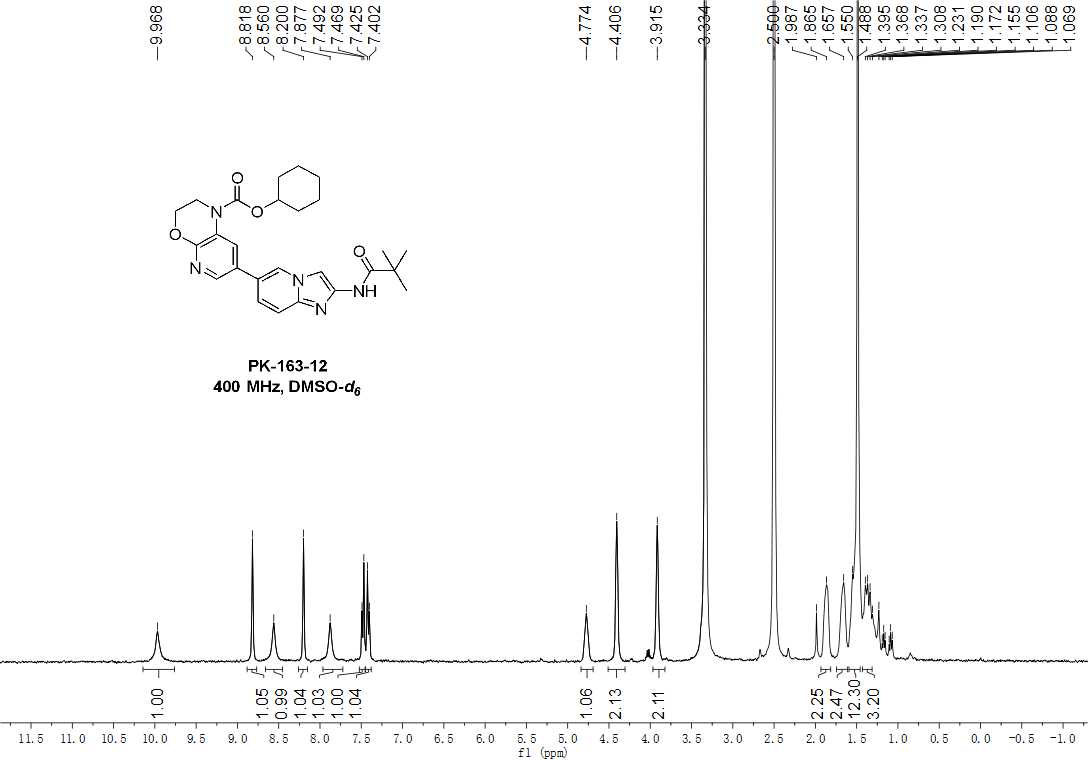


**Supplemental Figure 10**. ^1^H NMR spectra for **Zharp1-163-12**


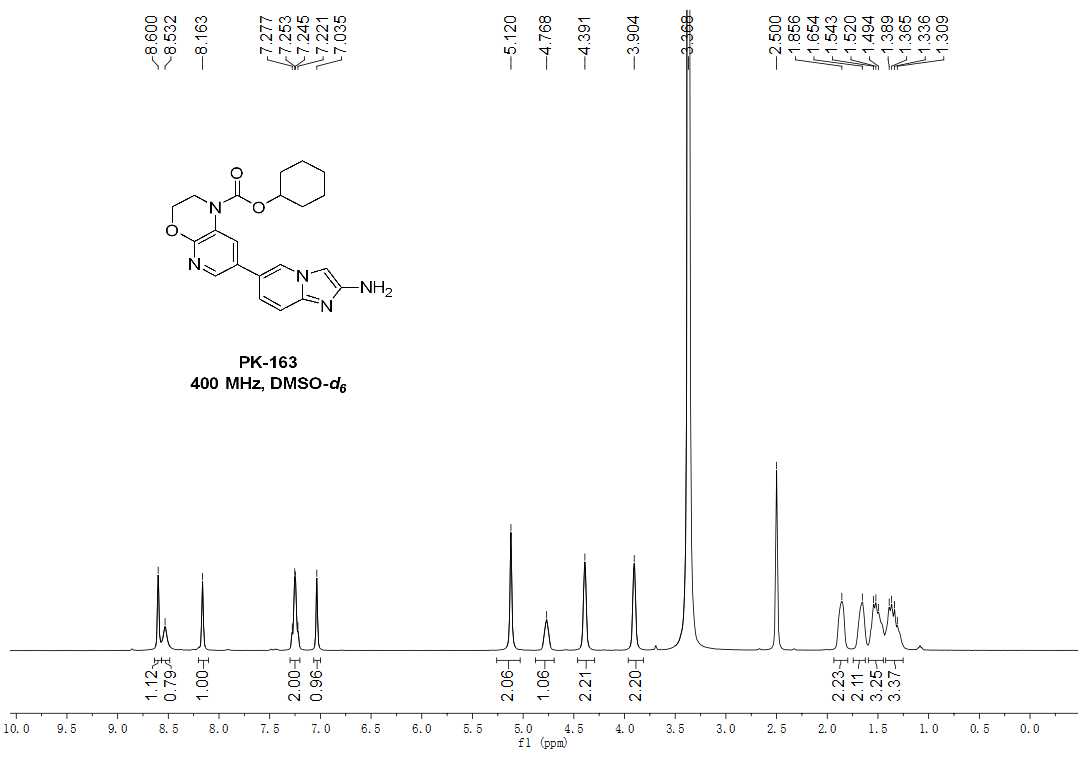


**Supplemental Figure 11**. ^1^H NMR spectra for **Zharp1-163**


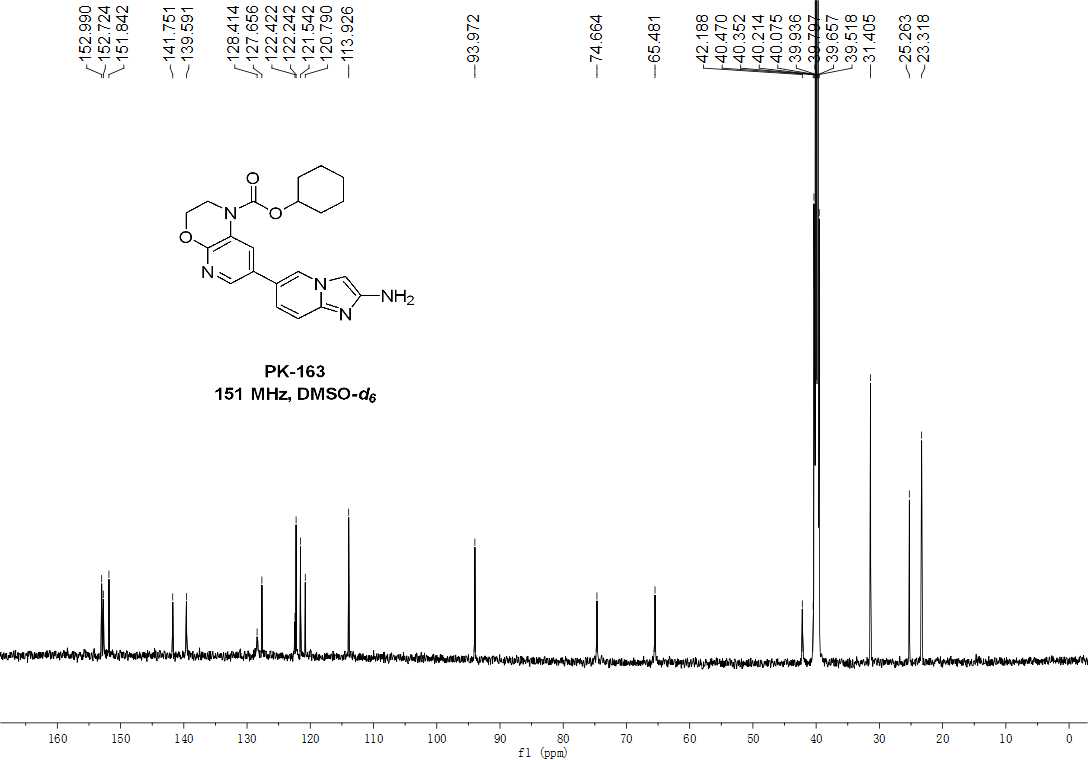


**Supplemental Figure 12**. ^13^C NMR spectra for **Zharp1-163**

**Supplemental Table 1. Kinase inhibitory activities of Zharp1-163 @1 μM against 81 human protein Kinases^a^**

| Kinase | Activity% | | Kinase | Activity% | |
| --- | --- | --- | --- | --- | --- |
|  | Data1 | Data2 |  | Data1 | Data2 |
| TRKB | 61.4 | 58.07 | DYRK1B | 16.55 | 9.23 |
| PIK3CA | 37.1 | 35.75 | IRAK4 | 8.12 | 17.2 |
| CLK1 | 38.13 | 33.19 | AurB | 8.89 | 13.38 |
| MLK1 | 21.18 | 32.72 | MET | 18.32 | 3.81 |
| TIE2 | 11.87 | 26.5 | p38α | 11.79 | 9.57 |
| GAK | 18.21 | 19.69 | PEK | 1.73 | 19.45 |
| ITK | 19.38 | 18.46 | HPK1 | 17.04 | 2.46 |
| CK1γ3 | 14.12 | 20.55 | AMPKα1/β1/γ1 | 12.1 | 6.96 |
| Erk2 | 17.88 | 10.28 | PKD2 | 11.99 | 6.79 |
| CHK1 | 16.8 | 10.43 | NEK1 | 4.63 | 13.42 |
| CHK2 | 15.77 | 1.85 | RSK1 | 6.09 | 5.81 |
| ABL1 | 6.71 | 9.11 | JNK1 | 6.2 | 5.09 |
| HGK | 4.09 | 11.54 | PIM1 | 3.88 | 7.34 |
| TGFβR1 | 7.16 | 7.76 | SYK | 5.12 | 4.56 |
| MARK4 | 14.33 | 0.49 | CDK5/p35NCK | 7.89 | 1.25 |
| WEE1 | 9.26 | 4.33 | GSK3β | 11.75 | -2.82 |
| CDK1/CycE1 | 5.77 | 6.95 | BMX | -3.03 | 11.34 |
| AXL | 10.89 | 1.24 | IKKα | 0.19 | 7.24 |
| QIK | 9.18 | 2.94 | ROCK1 | 2.59 | 4.57 |
| TGFβR2 | 7.99 | 3.92 | DCAMKL2 | 5.19 | 1.92 |
| HIPK1 | 8.39 | -1.36 | SGK | 2.39 | 0.81 |
| Erk7 | 5.82 | 0.29 | CDK2/CycE1 | 3.25 | -0.65 |
| MAP3K19 | -4.19 | 10.21 | FGFR1 | 2.7 | -0.57 |
| JAK3 | -1.37 | 7.33 | PDGFRα | -2.74 | 4.62 |
| CK1γ2 | -3.03 | 8.14 | TSSK1 | -0.22 | 2.05 |
| AKT1 | -1.95 | 6.27 | TAOK1 | 2.07 | -4.35 |
| CDK18/CycY | 4.89 | -0.64 | IRAK1 | 1.3 | -3.6 |
| MRCKβ | -0.71 | 4.66 | BRAF | 6.11 | -8.57 |
| DAPK1 | 9.17 | -5.62 | NEK2 | -4.5 | 1.76 |
| PKACα | -0.64 | 3.94 | EPHB4 | 1.63 | -4.43 |
| RIPK2 | -0.35 | -3.05 | BRSK2 | -6.15 | -3.32 |
| ALK4 | -1.47 | -2.74 | PKCα | -5.43 | -7.15 |
| PAK2 | -3.2 | -2.35 | IGF1R | -8.23 | -5.7 |
| SRC | -5.44 | -0.43 | ULK2 | -5.94 | -8.78 |
| p70S6K | -3.27 | -3.18 | EPHA2 | -8.55 | -9.14 |
| EGFR | -2.16 | -4.3 | CK1α | -4.65 | -16.63 |
| CK2α1/β | -12.2 | 5.04 | PLK2 | -2.91 | -19.68 |
| KDR | -2.59 | -4.74 | PLK1 | -5.06 | -18.01 |
| MARK1 | -11.47 | 4.11 | CaMK1α | -18.71 | -6.35 |
| MAP2K1 | -8.67 | 0.16 | LCK | -16.91 | -10.75 |
| MAPKAPK2 | -3.5 | -31.78 |  |  |  |

^a^ Compound **Zharp1-163** was tested at 1 μM in duplicate against 81 kinases using the RBC kinase panel service of Reaction Biology Corporation. All the ATP concentrations used were 10 μM. The 81 kinase panel did not include RIPK1. Full protocol details are as follows:

1. Compounds Preparation.
2. The compounds were serial diluted from 10 mM stock in DMSO.
3. Dilute the test compound to 0.1 mM (100 times the specified test concentration).The same dilution was used for the positive drugs in the experiment.
4. Compound screening
   1. Assay Buffer:

HTRF: ADP-Glo:

| **Reagent** | **Work Conc.** |
| --- | --- |
| 5× Buffer | 1× |
| MgCl_2_ | 5 mM |
| MnCl_2_ | 1 mM |
| SEB | 12.5 nM |
| DTT | 1 mM |
| ddH_2_O | / |

| **Reagent** | **Work Conc.** |
| --- | --- |
| Hepes | 50 mM |
| MgCl_2_ | 10 mM |
| Brij35 | 0.01% |
| EGTA | 1 mM |
| DTT | 2 mM |
| ddH_2_O | / |

2.2. HTRF Kinase Assay

a) 2× ATP & Substrate solution and 2× kinase & Metal solution were prepared using assay buffer.

b) Transfer 50 nL compound to 384 assay plate by Echo 655. After centrifugation, add 2.5 μL 2 × kinase/metal solution 384 assay plate, and incubate at 25℃ for 10 minutes.

c) 2.5μL of 2× Substrate & ATP solution were added to the well, and incubated at 25℃ for 60 minutes.

d) 2× XL665 & Antibody solution were prepared with detection buffer.

e) 5 μL of Kinase Detection Reagent was added to the well, and incubated for 60 minutes at 25℃.

f) The fluorescence signals of 620 nm (Cryptate) and 665 nm (XL665) were read by microtiter plate reader.

2.3 ADP-Glo Kinase Assay

a) 2× ATP & Substrate solution and 2× kinase & Metal solution were prepared using assay buffer

b) Transfer 40 nL compound to 384 assay plate by Echo 655. Add 2 μL of 2× kinase & Metal solution were mixed and incubated in a 384 assay plate for 10 minutes at 25℃.

c) 2 μL of 2× Substrate & ATP solution were added to the well, and incubated at 25℃ for 60 minutes.

d) 4 μL of ADP-Glo Reagent was added to the well, and incubated for 40 minutes at 25℃.

e) 8 μL of Kinase Detection Reagent was added to the well, and incubated for 40 minutes at 25℃.

f) The luminescence signals were recorded on microtiter plate reader.

3. Data Analysis

The readout value of reaction control (1% DMSO) was set as a 0% inhibition, and the readout value of background (10 µM Positive Control) was set as a 100% inhibition, then the percent inhibition of each test solution was calculated.% Inhibition was calculated as follow:

%Inhibition=100%-(compound-positive control)/ (negative control-positive control)*100%

positive control : The average data for the positive controls (10 μM)

negative control : The average data for negative controls (1% DMSO)
