# Supplementary material for: Discovery of Zharp1-163 as a dual inhibitor of ferroptosis and necroptosis for the treatment of inflammatory disorders and kidney injury
Source: Cell Death Discov. 2025 Aug 28;11:413. doi: 10.1038/s41420-025-02693-5 (PMC12394658; doi:10.1038/s41420-025-02693-5)
Supplement: Supplementary file 2 — Supplemental Material-Zharp1-163-Original Western Blots [file 41420_2025_2693_MOESM2_ESM.docx]

**Fig 2E**


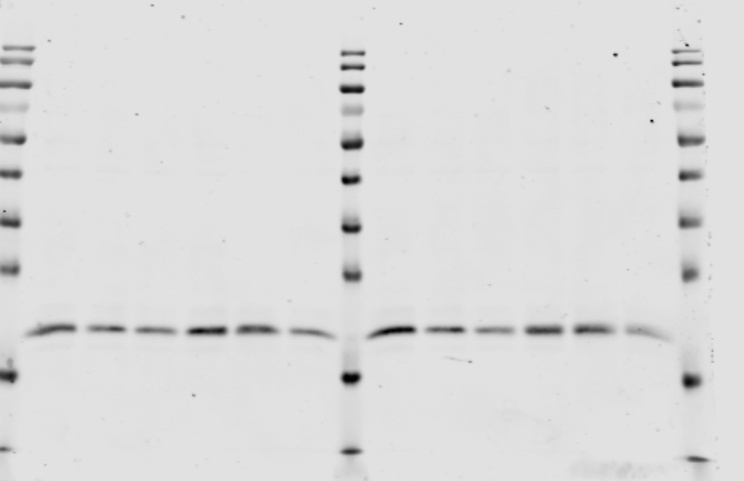


180

130

25

35

15

10

**GPX4**

70

100


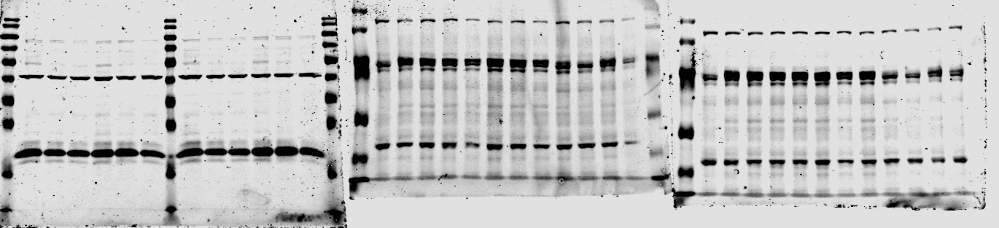


180

130

35

25

**β-actin**

40

55

70

100

40

55

**Fig 3A**

25

**RIPK1**


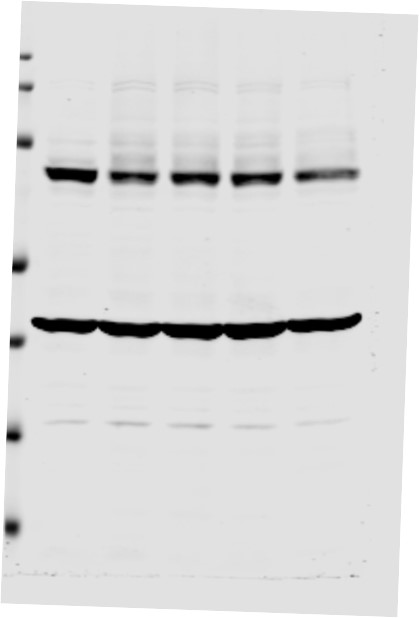


55

40

35

70

100

130

180


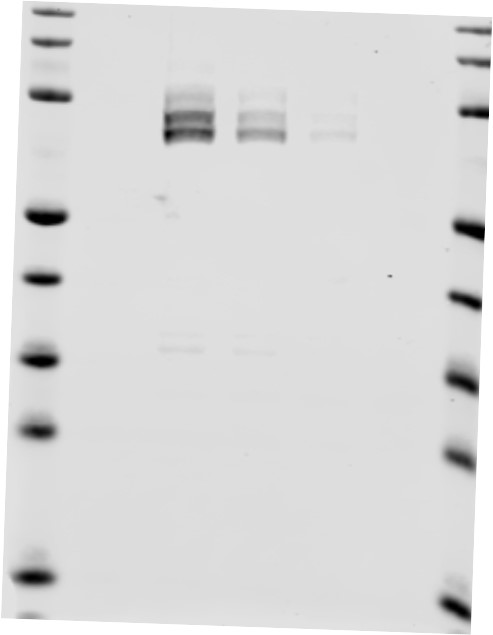


**p-RIPK1**

40

35

25

70

15

180

130

100

55


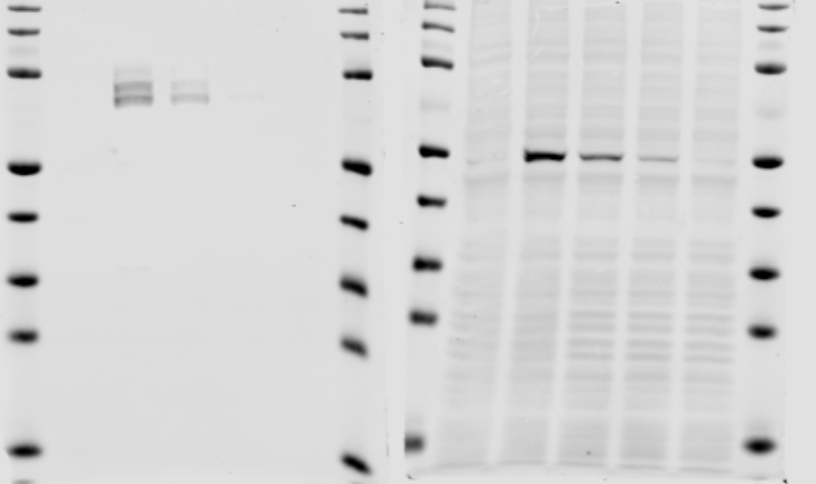


**p-MLKL**

55

40

35

25

70

100

15

130

180

130

25

**RIPK1**


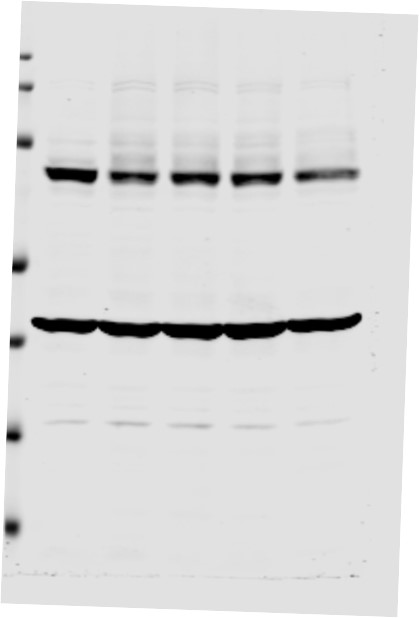


55

40

35

70

100

130

180


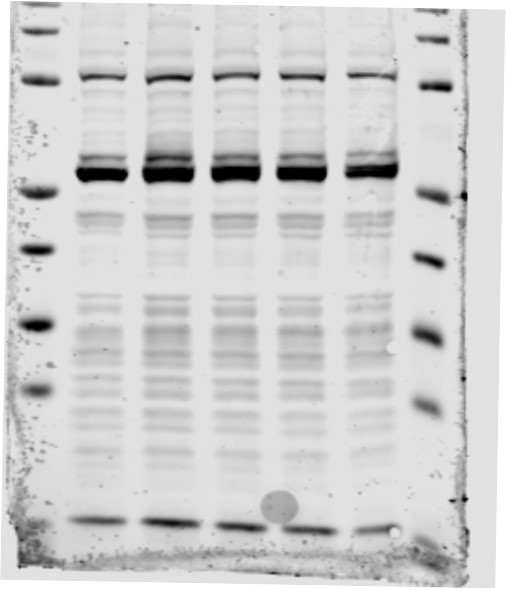


**RIPK3**

55

40

35

25

70

100

15

130

180


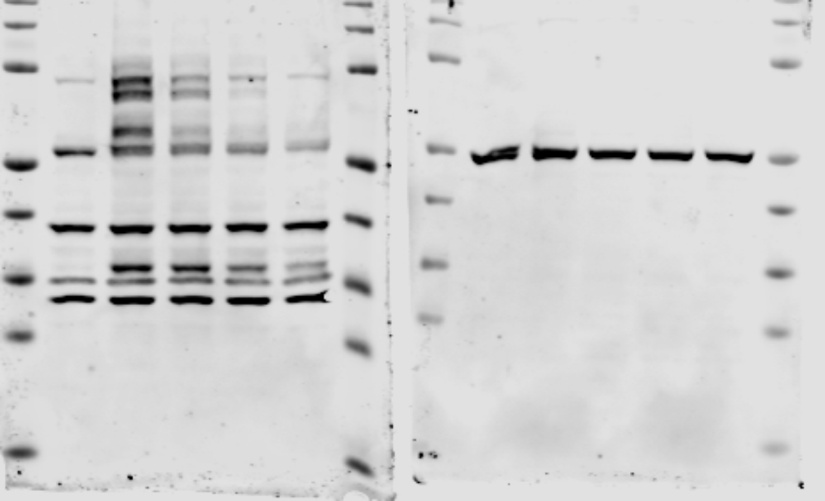


**p-RIPK3**

55

40

35

25

70

100

15


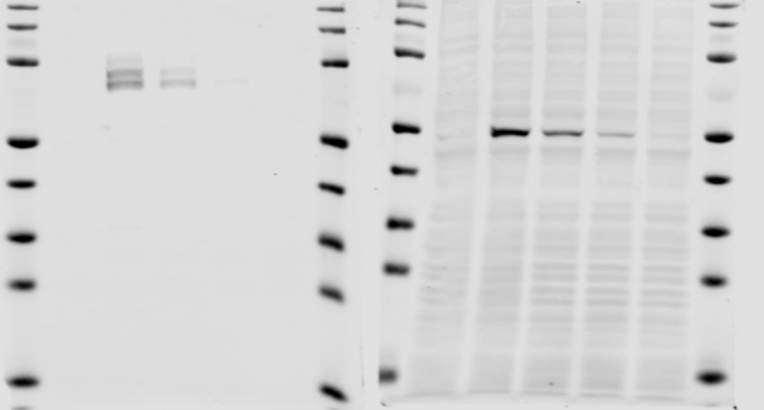


55

40

35

25

70

100

15

130

180

**p-MLKL**


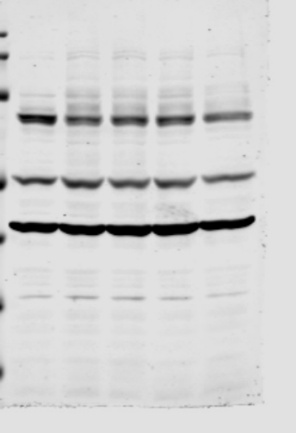


**MLKL**

25

55

40

35

70

100

130

180


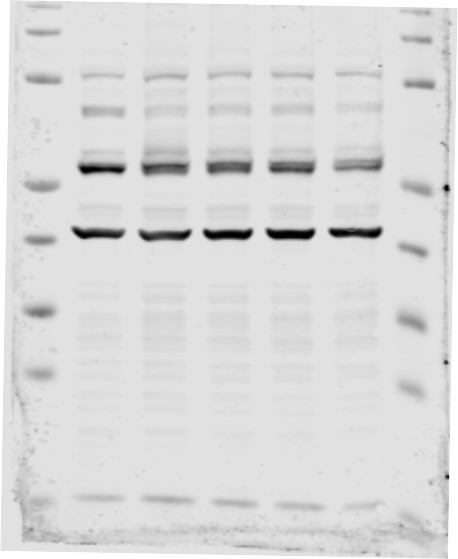


55

40

35

25

70

100

15

130

**β-actin**

**Fig 3B**


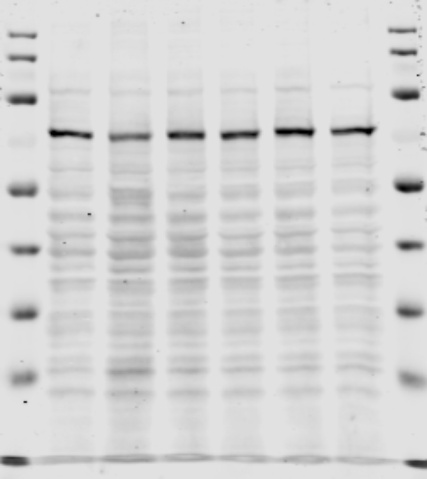


**RIPK1**

55

40

35

25

70

100

130

180


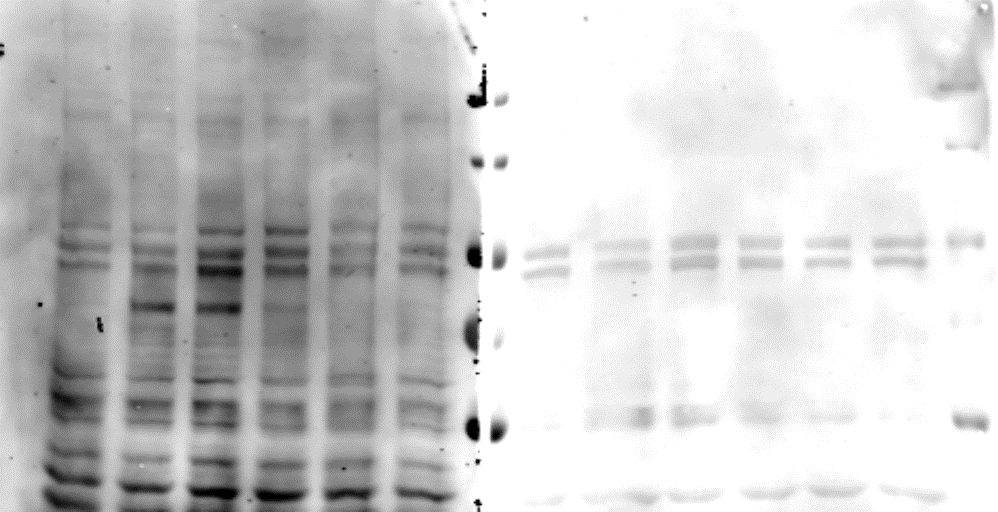


55

70

100

130

180

**p-RIPK1**


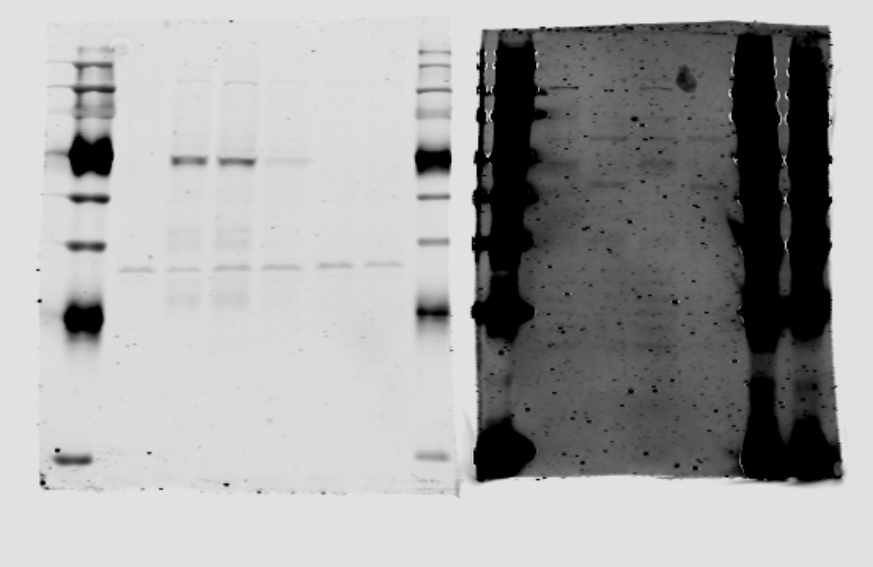


**p-RIPK3**

55

40

35

25

70

100

130

180

15


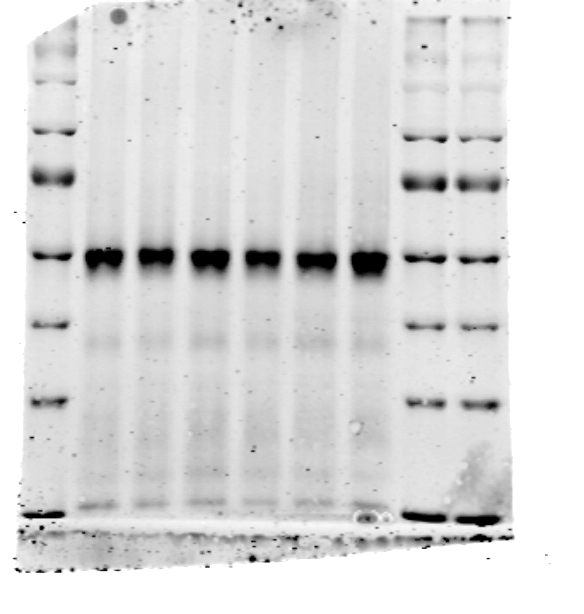


**RIPK3**

55

40

35

70

100

130

180


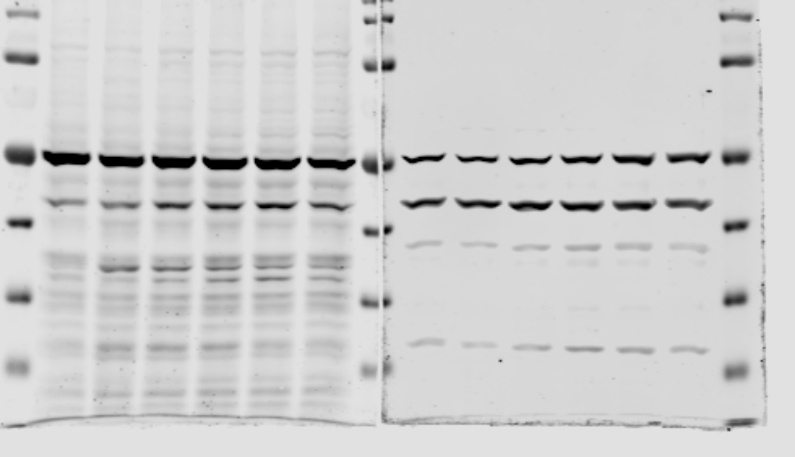


**MLKL**

55

40

35

70

100

130

25


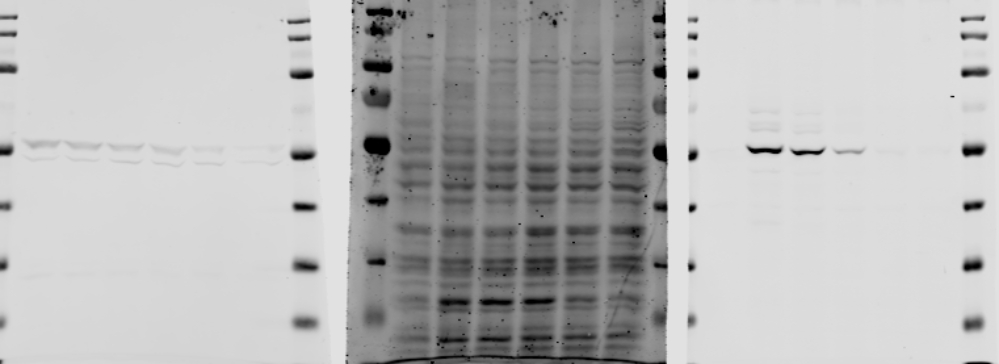


**p-MLKL**

55

40

35

25

70

100

130

180


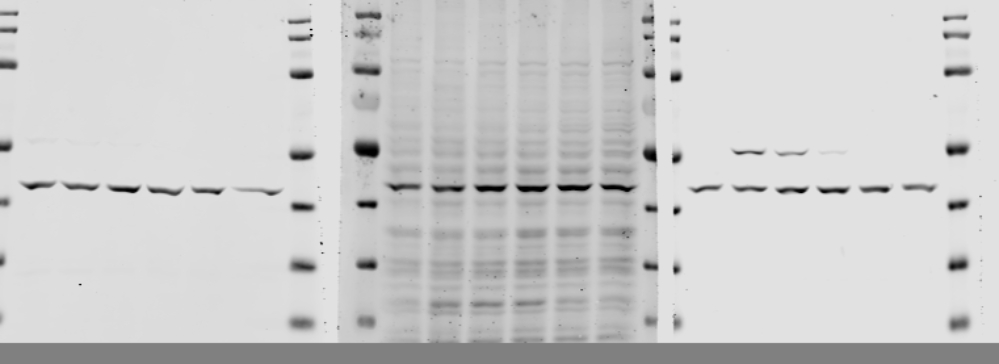


**β-actin**

55

40

35

70

100

130

180

25


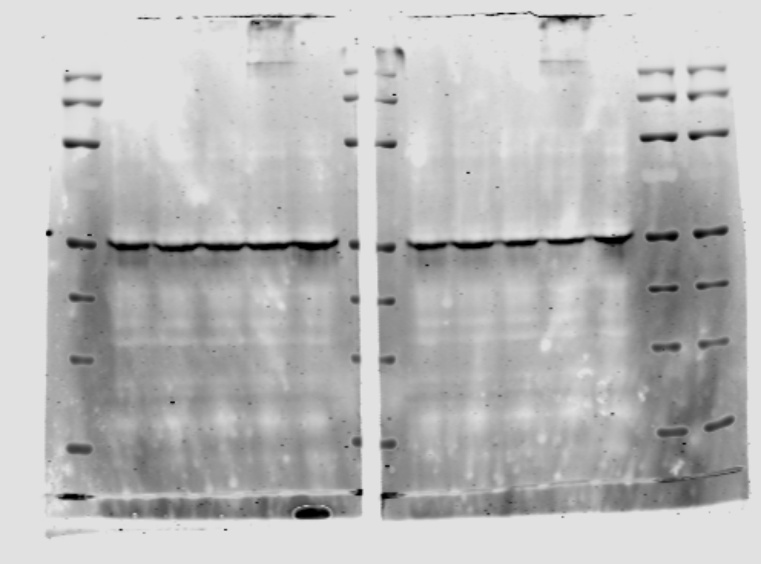


55

40

35

70

100

130

180

25

15

**MLKL**

**Oligomer**

**MLKL**

**Monomer**


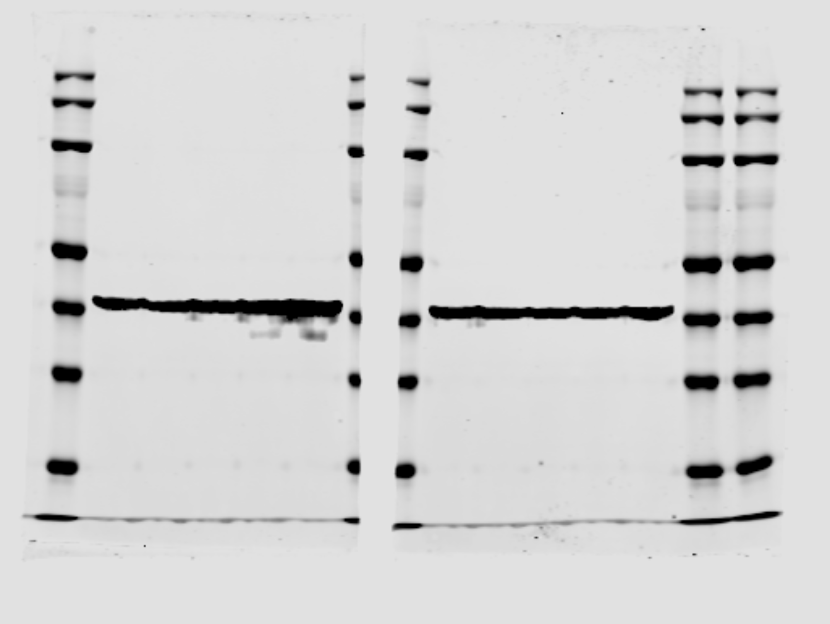


55

40

35

70

100

130

180

25

15

**β-actin**

**Fig 3G**
